# Screw analysis and experimental study of spatial noncircular gear compound transmission

**DOI:** 10.1371/journal.pone.0274575

**Published:** 2022-09-30

**Authors:** Yongquan Yu, Chao Lin

**Affiliations:** State Key Laboratory of Mechanical Transmission, Chongqing University, Chongqing, China; Beijing Institute of Technology, CHINA

## Abstract

Curve face gear pair and noncircular bevel gear pair are collectively referred to as spatial noncircular gear pair (SNCGP). Different from the traditional gear transmission with fixed shafts, spatial noncircular gear (SNCG) compound transmission can realize compound motion with variable transmission ratio between intersecting axes, and has a broad engineering application prospect. It includes two categories named speed reduction and speed increase. Based on the gear meshing theory, screw theory and calculus, a screw analysis method of SNCG compound transmission was proposed, which directly and comprehensively reflected the compound motion characteristics of rotation and axial movement. Firstly, a unified coordinate system was established. The screw geometric characteristics of compound motion were discussed, including instant screw axis, axode, pitch surface and its normal equidistant surface. The screw principle of compound motion was revealed. Then, according to this method, combined with the motion process of generator with different tooth profiles, the tooth surface of each SNCG was obtained. Finally, gear machining, tooth surface measurement, transmission ratio measurement and axial displacement measurement were carried out, which verified the correctness of this screw analysis method for SNCG compound transmission, and laid a foundation for further study and application.

## Introduction

Gear geometry and meshing theory are the basis of gear transmission. By using space vector and differential geometry as the theoretical basis and mathematical tools, many scholars have conducted research on the gear transmission with fixed shafts and established a complete basic theoretical system, covering meshing theory, design, tooth surface geometry and manufacturing [1–4]. Due to its geometrical intuition and algebraic abstraction in the description of spatial motion and algebraic operations, screw theory has become the most popular mathematical tool in mechanics and kinematics in the 21st century, and it has been applied by many theoretical kineticists and roboticists [5–7]. In view of the intuitive geometric description and integrated algebraic form of screw algebra, it can also be used to study the gear transmission, which has the characteristics of simple form and rich connotation. Hunt [8] used screw theory and Lie algebra to study gear transmission, and established the kinematics and dynamics model of the transmission system. Phillips [9] used screw theory as a mathematical tool to study the spatial involute gear transmission with skew axes. Based on the screw theory, Dooner et al. [10, 11] proposed the three laws of spatial gear meshing, unified the basic theory of spatial gear design and plane gear design, and then established a complete kinematic geometry theory of gearing. Zheng et al. [12] used screw theory to study the forming process of noncircular bevel gear transmission with free tooth profile. Hu et al. [13] studied the mathematical model of of curve face gear pair, analyzed the meshing characteristics of compound transmission with screw theory, and this gear pair can be applied to the focusing mechanism.

Noncircular gear is a mechanism that can realize a variable transmission ratio between a gear pair [14]. It is widely used in agriculture, textile and instruments, because of the advantages of low economic cost, compact structure, accurate transmission ratio, high efficiency, and high precision. In the 14th century, astronomical clocks began to use high precision noncircular gear mechanism [15]. After the 20th century, many scholars have carried out research on noncircular gear and made great progress in basic theory and engineering application. Litvin et al. [14] carried out systematic study on the design and manufacture of noncircular gear. Liu et al. [16] studied the application of a noncircular gear train in the generation of specific trajectory curve. Mundo et al. [17, 18] studied the tooth surface design method, motion characteristics, and application occasions of noncircular gear planetary transmission, also studied the noncircular gear five-link mechanism, and optimized the connecting rod curve of the traditional five-link mechanism based on the noncircular gear. Ottaviano et al. [19] studied the kinematic characteristics of noncircular gear and cam function generator by numerical calculation and experiment. Alexandru et al. [20] deeply analyzed the characteristics and geometric design method of variable transmission ratio steering gear. According to relevant knowledge, Ding et al. [21] proposed an enabling multi-sensor fusion-based longitudinal vehicle speed estimator for four-wheel-independently-actuated electric vehicles and conducted some relevant researches [22, 23]. Zheng et al. [24] proposed a new face milling method, which can be used for the design and manufacture of noncircular cylindrical gears. Yu et al. [25] carried out a simulation and experimental study on the surface topography and machining surface quality of noncircular gear in ball end milling. Based on the research of noncircular gear, scholars have carried out some innovative researches. For example, noncircular bevel gear has been studied by scholars, involving the mathematical model, tooth surface geometric design and manufacturing and other aspects [26–29]. Lin et al. [30] creatively proposed a new type of curve face gear pair, which can also realize a variable transmission ratio between intersecting axis, and carried out in-depth researches [31–33].

At present, there are few researches on the gear compound transmission. According to the gear meshing theory, Lin et al. [34–37] proposed some compound transmission gear pairs, and separately carried out the geometric design and motion analysis from the aspect of space vector.

However, the above relevant researches are all aimed at a single compound transmission respectively, and are not universal and comprehensive for different types of SNCG compound transmissions. Meanwhile, compared with the space vector representation, the screw representation can more intuitively and clearly describe the feature of compound transmission, which has rotation and axial movement simultaneously. So far, the basic theory of SNCG compound transmission is not complete. Therefore, it is necessary to propose a general analysis method for SNCG compound transmission, and the new proposed screw analysis method can directly and comprehensively reflect the compound motion characteristics of rotation and axial movement. Firstly, according to the gear meshing theory, screw theory and calculus, a unified coordinate system was established. The screw geometric characteristics of compound motion were discussed, and the screw principle of compound motion was revealed. Then, combined with the tooth surface equation and motion process of generator with different tooth profiles, the tooth surface of each SNCG was obtained. Finally, gear machining, tooth surface measurement, transmission ratio measurement and axial displacement measurement were carried out, which verified the rationality and correctness of this screw analysis method. Based on the compound transmission characteristic, this SNCG compound transmission can be used in the fields of percussion drill and plunger pump, and the screw analysis method can provide some theoretical guidance for the design and application of similar mechanisms.

## Screw analysis of SNCG compound transmission

### Composition of SNCG compound transmission

**Fig 1:**
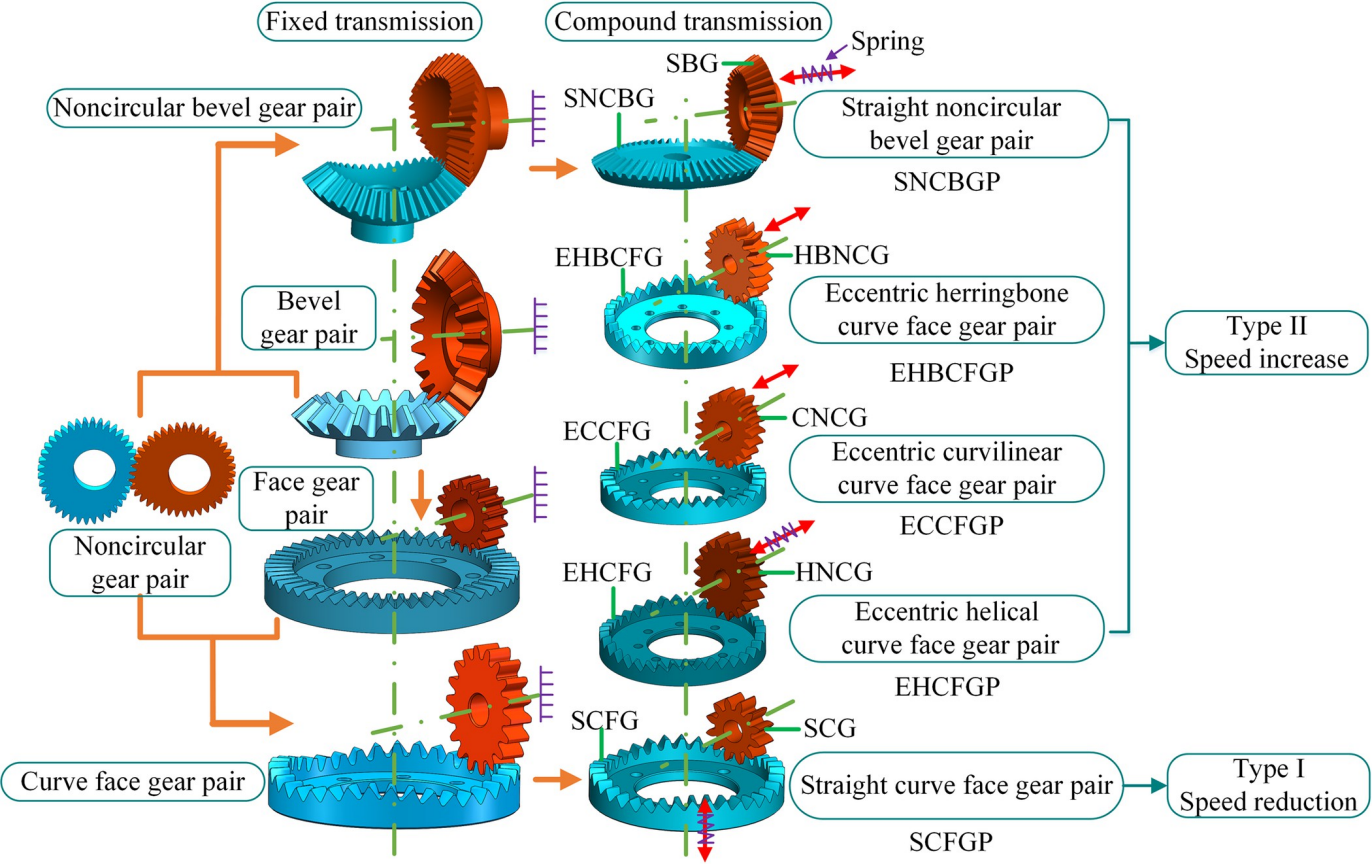
Composition of SNCG transmission.

Gear transmission is generally divided into the fixed type and the compound type. The fixed type means that the output shaft only rotates without moving, and the compound type means that the output shaft rotates and axially reciprocates simultaneously, as shown in Fig 1. SNCG compound transmission includes two categories named speed reduction (type I) and speed increase (type II), with a total of five subcategories. Type I is the straight curve face gear pair (SCFGP), which is composed of a straight cylindrical gear (SCG) and a straight curve face gear (SCFG). Type II includes the following four gear pairs: the eccentric helical curve face gear pair (EHCFGP) composed of a helical noncircular gear (HNCG) and a eccentric helical curve face gear (EHCFG), the eccentric curvilinear curve face gear pair (ECCFGP) composed of a curvilinear noncircular gear (CNCG) and a eccentric curvilinear curve face gear (ECCFG), the eccentric herringbone curve face gear pair (EHBCFGP) composed of a herringbone noncircular gear (HBNCG) and a eccentric herringbone curve face gear (EHBCFG), and the straight noncircular bevel gear pair (SNCBGP) composed of a straight bevel gear (SBG) and a straight noncircular bevel gear (SNCBG). Besides, the axial reciprocation of the driven gear of SCFGP, EHCFGP and SNCBGP is realized by the elastic force of the spring, so this case is called force recovery. The axial reciprocation of the driven gear of ECCFGP and EHBCFGP is realized by the shape of gear tooth, so this case is called shape recovery. The double-headed arrow indicates the axial reciprocating motion of the driven gear.

The Abbreviations of relevant gear pairs are shown in Table 1.

**Table 1:** Abbreviations of gear pairs.

Names		Abbreviations	
Spatial noncircular gear pair	Spatial noncircular gear	SNCGP	SNCG
Straight curve face gear pair	Straight cylindrical gear	SCFGP	SCG
	Straight curve face gear		SCFG
Eccentric helical curve face gear pair	Helical noncircular gear	EHCFG	HNCG
	Eccentric helical curve face gear		EHCFG
Eccentric curvilinear curve face gear pair	Curvilinear noncircular gear	ECCFGP	CNCG
	Eccentric curvilinear curve face gear		ECCFG
Eccentric curvilinear curve face gear pair	Herringbone noncircular gear	EHBCFGP	HBNCG
	Eccentric herringbone curve face gear		EHBCFG
Straight noncircular bevel gear pair	Straight bevel gear	SNCBGP	SBG
	Straight noncircular bevel gear		SNCBG

### Unified coordinate system and pitch curve

#### Unified coordinate system

**Fig 2:**
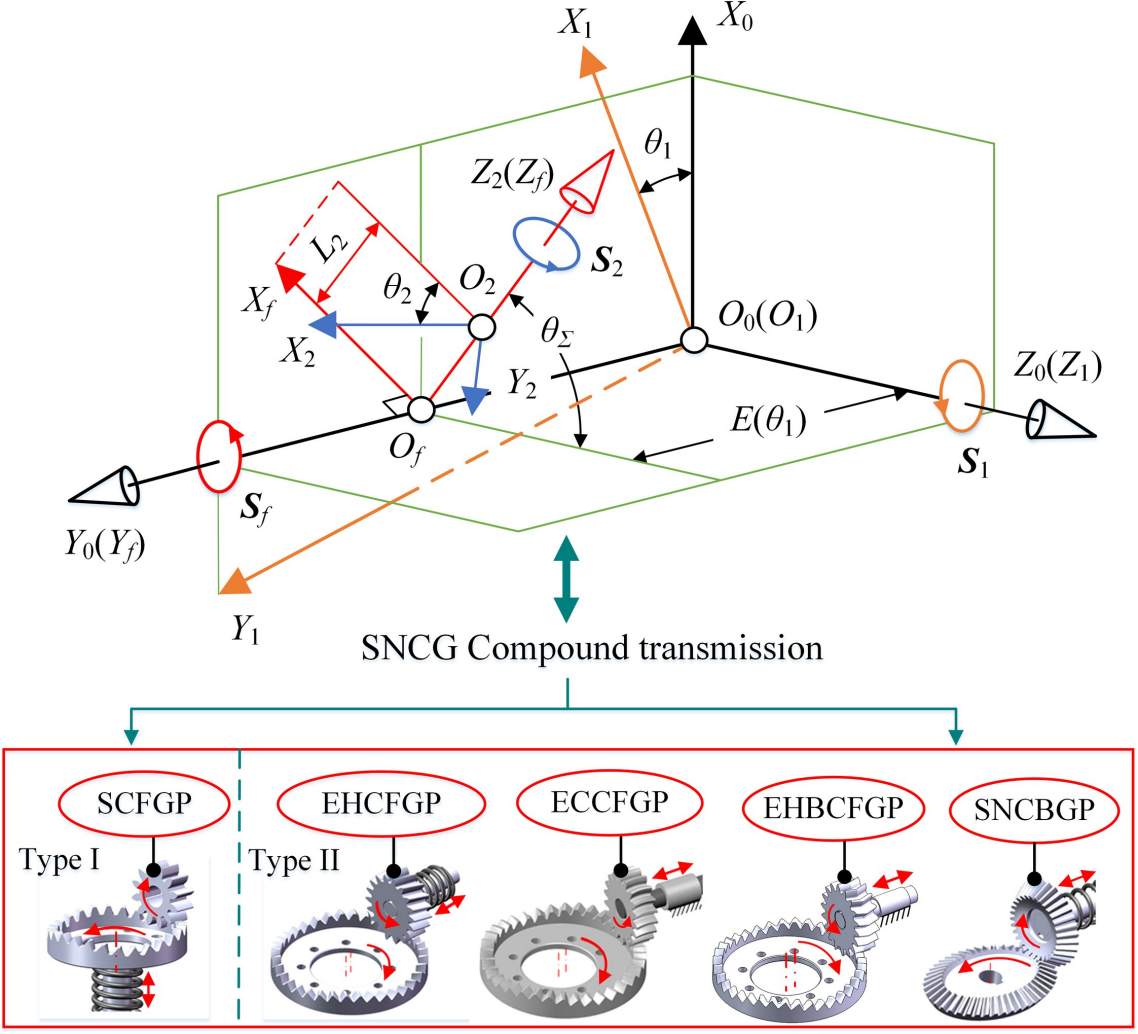
Unified coordinate system of SNCG compound transmission.

According to the form of SNCG compound transmission, a unified coordinate system was established, as shown in Fig 2. ***S***_1_, ***S***_2_ and ***S***_*f*_ are the screw of gear 1, gear 2 and axis of gear 2 respectively. *θ*_1_ and *θ*_2_ are the rotation angles of gear 1 and gear 2, respectively. *S*_0_(*O*_0_‒*X*_0_*Y*_0_*Z*_0_), abbreviated as *S*_0_, is the global coordinate system, which keeps still. *S*_1_(*O*_1_‒*X*_1_*Y*_1_*Z*_1_), abbreviated as *S*_1_, is the follow-up coordinate system of gear 1. *O*_1_*Z*_1_ is the rotation axis of gear 1 and coincides with *O*_0_*Z*_0_. *S*_2_(*O*_2_–*X*_2_*Y*_2_*Z*_2_), abbreviated as *S*_2_, is the follow-up coordinate system of gear 2. *S*_*f*_ (*O*_*f*_–*X*_*f*_*Y*_*f*_*Z*_*f*_), abbreviated as *S*_*f*_, is the follow-up coordinate system of the axis of gear 2 and moves with axis *O*_2_*Z*_2_. *O*_*f*_*Y*_*f*_ coincides with *O*_0_*Y*_0_. *O*_*f*_*Z*_*f*_ coincides with *O*_2_*Z*_2_. *θ*_Σ_ is the rotation angle of *S*_*f*_ around *O*_0_*Y*_0_ with respect to *S*_0_. *E*(*θ*_1_) is the length of *O*_0_*O*_*f*_.

The relationship between *θ*_1_ and *θ*_2_ is

$$\theta_{2}=\int_{0}^{\theta_{1}}i_{21}(\theta )d\theta$$ (1)

Where, *i*_21_(*θ*) = *ω*_2_/*ω*_1_ is the transmission ratio. *ω*_1_ and *ω*_2_ are the angular velocities of gears 1 and 2, respectively.

Axial displacement of gear 2 is

$$L_{2}(\theta_{2})=\int_{0}^{\theta_{2}}h_{2}(\theta )d\theta =\int_{0}^{\theta_{1}}h_{2}(\theta )i_{21}(\theta )d\theta$$ (2)

Where, *h*_2_ is the pitch of ***S***_2_.

For the SNCG compound transmission, *O*_1_*Z*_1_ and *O*_2_*Z*_2_ intersect vertically, that is, *θ*_Σ_ = π/2,*E*(*θ*_1_) = 0. The transformation matrix from *S*_1_ to *S*_2_ is

$$M_{21}=M_{2f}M_{f0}M_{01}=[\begin{array}{cccc} sin\theta_{1}\sin\theta_{2} & \cos\theta_{1}\sin\theta_{2} & -\cos\theta_{2} & 0\\ \cos\theta_{2}sin\theta_{1} & \cos\theta_{1}\cos\theta_{2} & \sin\theta_{2} & 0\\ \cos\theta_{1} & -sin\theta_{1} & 0 & -L_{2}(\theta_{2})\\ 0 & 0 & 0 & 1 \end{array}]$$ (3)

Where, ***M***_2*f*_, ***M***_*f*0_, ***M***_01_ are the transformation matrices from *S*_*f*_ to *S*_2_, *S*_0_ to *S*_*f*_ and *S*_1_ to *S*_0_ respectively.


$$M_{2f}=[\begin{array}{cccc} \cos\theta_{2} & \sin\theta_{2} & 0 & 0\\ -\sin\theta_{2} & \cos\theta_{2} & 0 & 0\\ 0 & 0 & 1 & -L_{2}(\theta_{2})\\ 0 & 0 & 0 & 1 \end{array}],M_{f0}=[\begin{array}{cccc} 0 & 0 & -1 & 0\\ 0 & 1 & 0 & 0\\ 1 & 0 & 0 & 0\\ 0 & 0 & 0 & 1 \end{array}],M_{01}=[\begin{array}{cccc} \cos\theta_{1} & -\sin\theta_{1} & 0 & 0\\ \sin\theta_{1} & \cos\theta_{1} & 0 & 0\\ 0 & 0 & 1 & 0\\ 0 & 0 & 0 & 1 \end{array}]$$ (4)

The shape of pitch curve and the axial displacement have an important influence on the realization of compound transmission and the following studies were carried out for type I and type II respectively.

#### Pitch curve of type I

**Fig 3:**
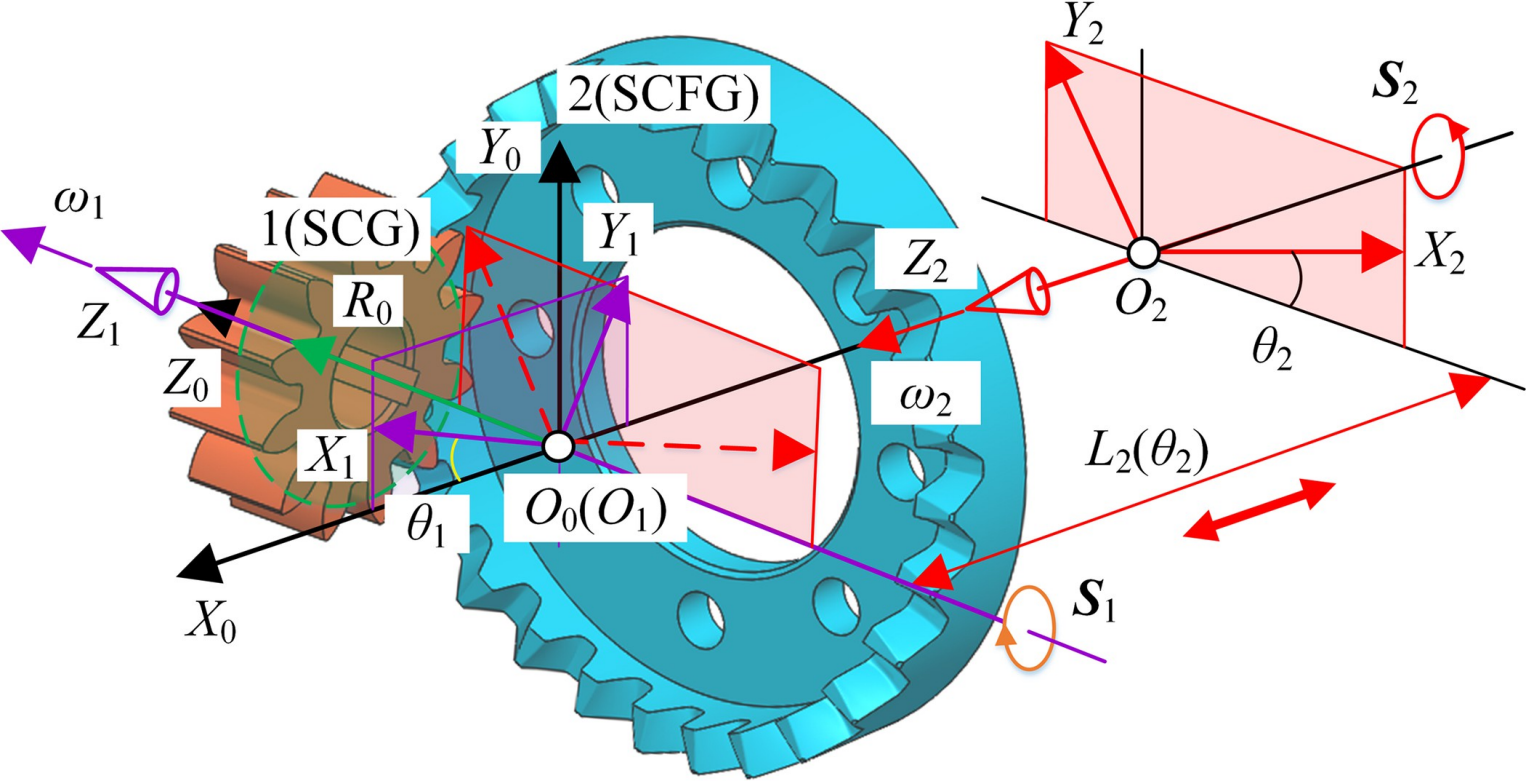
Coordinate system of type I.

Type I is the SCFGP. According to the unified coordinate system in Fig 2, the coordinate system of type I was established, as shown in Fig 3. The corresponding parameters have the same meaning as before.

The equation of the pitch curve of SCG in *S*_1_ is $r_{1}={\left[-r_{1}\cos\theta_{1},r_{1}\sin\theta_{1},R_{0},1\right]}^{T}$. *r*_1_ is the radius of the pitch circle of SCG. *R*_0_ is the distance between the plane where the pitch curve ***r***_1_ is located and plane *X*_0_*O*_0_*Y*_0_. The equation of the pitch curve of ECCFG in *S*_2_ can be expressed as

$$r_{2}=M_{21}r_{1}={\left[\begin{array}{cccc} -R_{0}\cos\theta_{2} & R_{0}\sin\theta_{2} & -r_{1}-L_{2}(\theta_{2}) & 1 \end{array}\right]}^{T}$$ (5)

According to the pure rolling relationship between the pitch curve of SCG and the pitch curve of ECCFG, we have

$$r_{1}\theta_{1}=\int_{0}^{\theta_{2}}\sqrt{R_{0}^{2}+{\left[dL_{2}(\theta )/d\theta \right]}^{2}}d\theta$$ (6)

Then the transmission ratio can be expressed as

$$i_{21}(\theta_{2})=\frac{\omega_{2}}{\omega_{1}}=\frac{d\theta_{2}}{d\theta_{1}}=\frac{r_{1}}{\sqrt{R_{0}^{2}+{\left[dL_{2}(\theta )/d\theta \right]}^{2}}}$$ (7)

**Fig 4:**
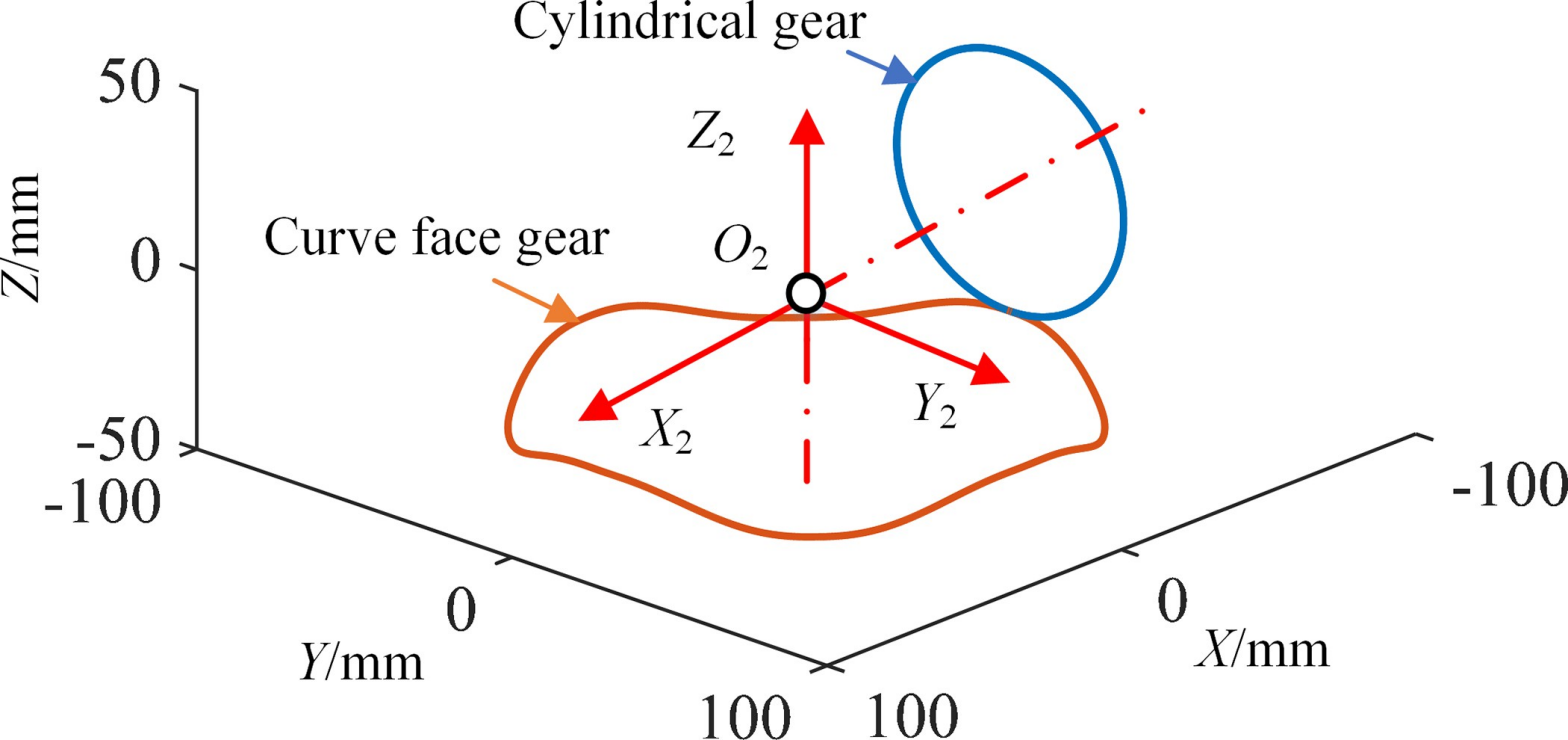
Pitch curves of SCFGP.

By changing *L*_2_, different compound transmissions can be realized. When *L*_2_ takes the expression in Eq (8) and the parameters in Table 2, the pitch curves of SCFGP can be obtained, as shown in Fig 4.


$$L_{2}(\theta_{2})=\{\begin{array}{l} -L_{0}(\frac{10\theta_{2}^{3}}{\theta_{a}^{3}}-\frac{15\theta_{2}^{4}}{\theta_{a}^{4}}+\frac{6\theta_{2}^{5}}{\theta_{a}^{5}}),\theta_{2}\in [0,\theta_{a}]\\ -L_{0}+L_{0}(\frac{10\theta_{2}^{3}}{\theta_{b}^{3}}-\frac{15\theta_{2}^{4}}{\theta_{b}^{4}}+\frac{6\theta_{2}^{5}}{\theta_{b}^{5}}),\theta_{2}\in [\theta_{a},\theta_{T}] \end{array}$$ (8)

**Table 2:** Pitch curve parameters of SCFGP.

*L*_0_	*n*_2_	*R*_0_	*θ*_*a*_	*r*_1_
10mm	2	69.2mm	π/4	36mm

Where, *L*_0_ is the amplitude of axial displacement, *θ*_*T*_ = 2π/*n*_2_ is the cycle of SCFG, *n*_2_ is the order of SCFG, *θ*_*a*_ is the corresponding rotation angle when *L*_2_ is *L*_0_. *θ*_*b*_ = *θ*_*T*_−*θ*_*a*_ and *θ*_*b*_ = *θ*_*T*_/2. By changing *θ*_*a*_ and *θ*_*b*_, the asymmetry of compound transmission can be achieved.

As can be seen from Fig 4, the pitch curve of SCG is a plane circle, the pitch curve of SCFG is a cylindrical curve with radius *R*_0_, and the coordinate value *Z*_2_ is related to *L*_2_. Specially, when *r*_1_ is a function of *θ*_1_, that is, SCG becomes a noncircular gear, and *L*_2_ is 0, this transmission becomes the fixed type.

#### Pitch curve of type II

**Fig 5:**
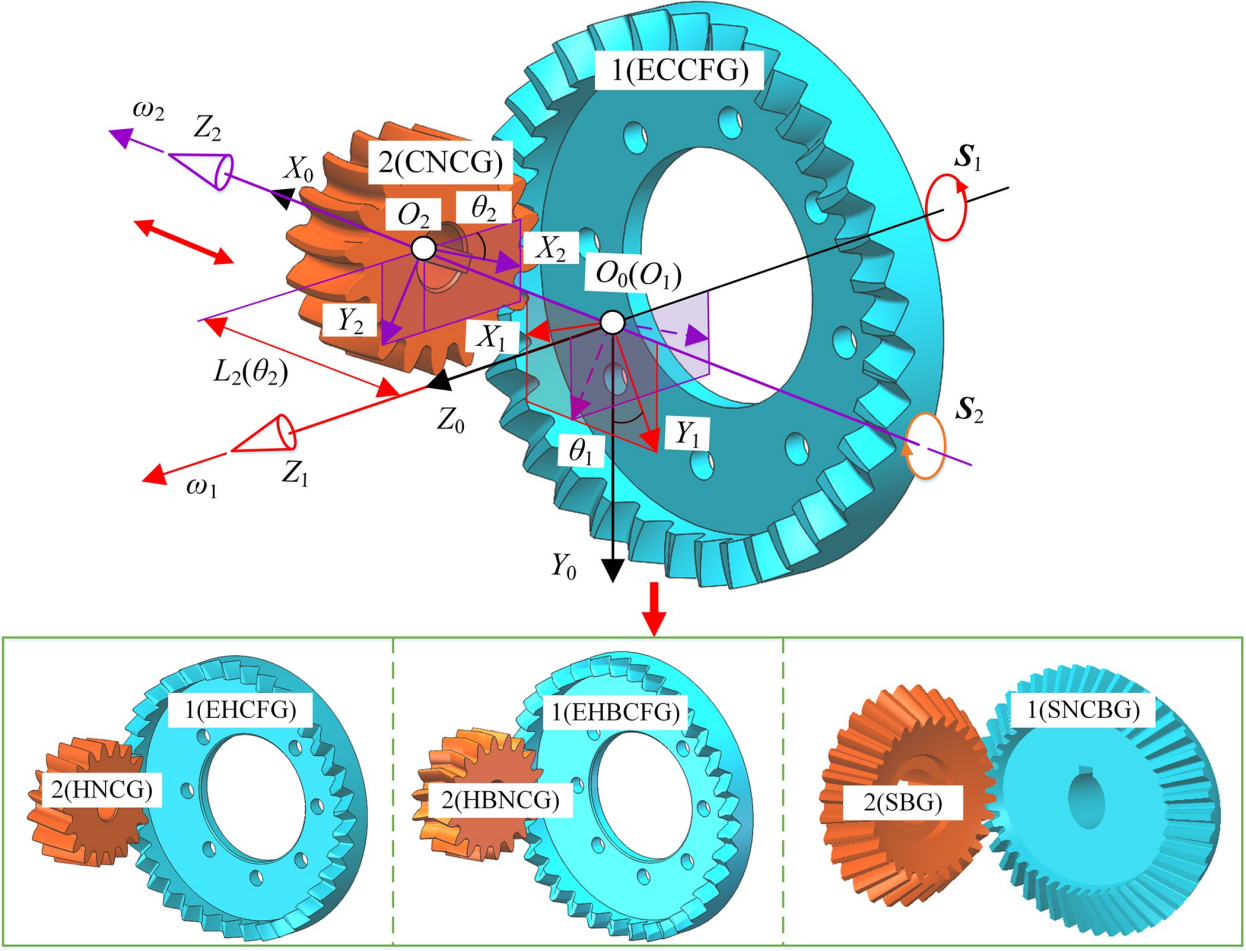
Coordinate system of type II.

Based on the unified coordinate system in Fig 2, the coordinate system of type II was established, as shown in Fig 5. The corresponding parameters have the same meaning as before.

#### (1) Eccentric curve face gear pair (ECFGP)

**Fig 6:**
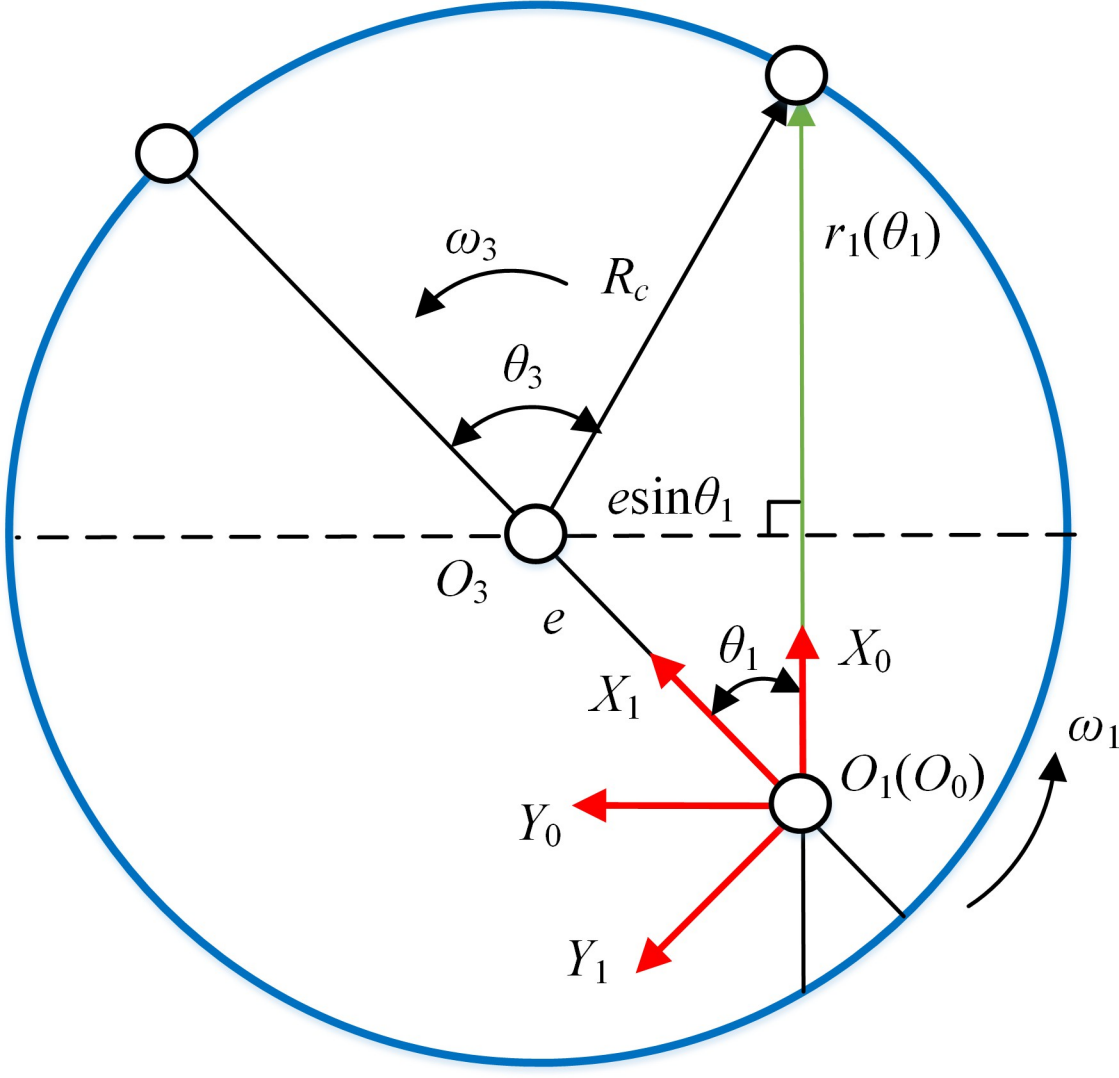
Projection of the pitch curve of ECFG on plane *X*_1_*O*_1_*Y*_1_.

ECFGP includes EHCFGP, ECCFGP and EHBCFGP. The compound motion of noncircular gear (NCG) is realized by the eccentricity of the eccentric curve face gear (ECFG). Assuming the equation of the pitch curve of ECFG in *S*_1_ is $r_{1}={\left[r_{1}(\theta_{1})\cos\theta_{1},-r_{1}(\theta_{1})\sin\theta_{1},-r_{2}(\theta_{2}),1\right]}^{T}$. *r*_1_(*θ*_1_) is the distance between the projection of the pitch curve of ECFG on plane *X*_1_*O*_1_*Y*_1_ and point *O*_1_, as shown in Fig 6. *r*_2_(*θ*_2_) is the coordinate value of the meshing point on axis *O*_1_*Z*_1_. Then the pitch curve of NCG in *S*_2_ can be expressed as

$$r_{2}=M_{21}r_{1}={\left[\begin{array}{cccc} r_{2}(\theta_{2})\cos\theta_{2} & -r_{2}(\theta_{2})\sin\theta_{2} & r_{1}(\theta_{1})-L_{2}(\theta_{2}) & 1 \end{array}\right]}^{T}$$ (9)

Fig 6 is the projection of the pitch curve of ECFG on plane *X*_1_*O*_1_*Y*_1_. *e* is the eccentricity. When *e* is 0, that is, the rotation center is *O*_3_ and ECFG is called the auxiliary gear 3. During the transmission process, NCG can be regarded as meshing with ECFG and gear 3 at the same time. *θ*_3_, *ω*_3_ and *R*_*c*_ are the rotation angle, angular velocity and radius of gear 3, respectively.

From the geometric relationship in Fig 6, we can get

$$\left\{ \begin{array}{l} r_{1}(\theta_{1})=\sqrt{R_{c}^{2}-e^{2}\sin^{2}\theta_{1}}+e\cos\theta_{1}=r_{1}(\theta_{3})=\sqrt{R_{c}^{2}+e^{2}-2eR_{c}\cos(\pi -\theta_{3})}\\ L_{2}(\theta_{2})=r_{1}(\theta_{1})-r_{1}(0),\theta_{1}=\left\{ \begin{array}{l} \arccos\frac{r_{1}^{2}(\theta_{3})+e^{2}-R_{c}^{2}}{2er_{1}(\theta_{3})},\theta_{3}\in [0,\pi ]\\ 2\pi -\arccos\frac{r_{1}^{2}(\theta_{3})+e^{2}-R_{c}^{2}}{2er_{1}(\theta_{3})},\theta_{3}\in [\pi ,2\pi ] \end{array}\right. \end{array}\right.$$ (10)

Then the pitch curve of NCG in *S*_2_ can be expressed as $r_{2}={\left[r_{2}(\theta_{2})\cos\theta_{2},-r_{2}(\theta_{2})\sin\theta_{2},r_{1}(0),1\right]}^{T}$. The transmission ratios between ECFG and gear 3, NCG and gear 3 are respectively

$$\left\{ \begin{array}{l} i_{13}=\omega_{1}/\omega_{3}=d\theta_{1}/d\theta_{3}\\ i_{23}=\omega_{2}/\omega_{3}=R_{c}/r_{2}(\theta_{2}) \end{array}\right.$$ (11)

Then we can get the transmission ratio between ECFG and NCG by

$$i_{21}=\frac{i_{23}}{i_{13}}=\{\begin{array}{l} \frac{r_{1}(\theta_{3})\sqrt{\left[4e^{2}r_{1}^{2}(\theta_{3})-{\left(r_{1}^{2}(\theta_{3})+e^{2}-R_{c}^{2}\right)}^{2}][R_{c}^{2}+e^{2}+2eR_{c}\cos(\theta_{3})\right]}}{er_{2}(\theta_{2})\sin\theta_{3}(r_{1}^{2}(\theta_{3})-e^{2}+R_{c}^{2})},\theta_{3}\in [0,\pi ]\\ \frac{-r_{1}(\theta_{3})\sqrt{\left[4e^{2}r_{1}^{2}(\theta_{3})-{\left(r_{1}^{2}(\theta_{3})+e^{2}-R_{c}^{2}\right)}^{2}][R_{c}^{2}+e^{2}+2eR_{c}\cos(\theta_{3})\right]}}{er_{2}(\theta_{2})\sin\theta_{3}(r_{1}^{2}(\theta_{3})-e^{2}+R_{c}^{2})},\theta_{3}\in [\pi ,2\pi ] \end{array}$$ (12)

#### (2) SNCBGP

**Fig 7:**
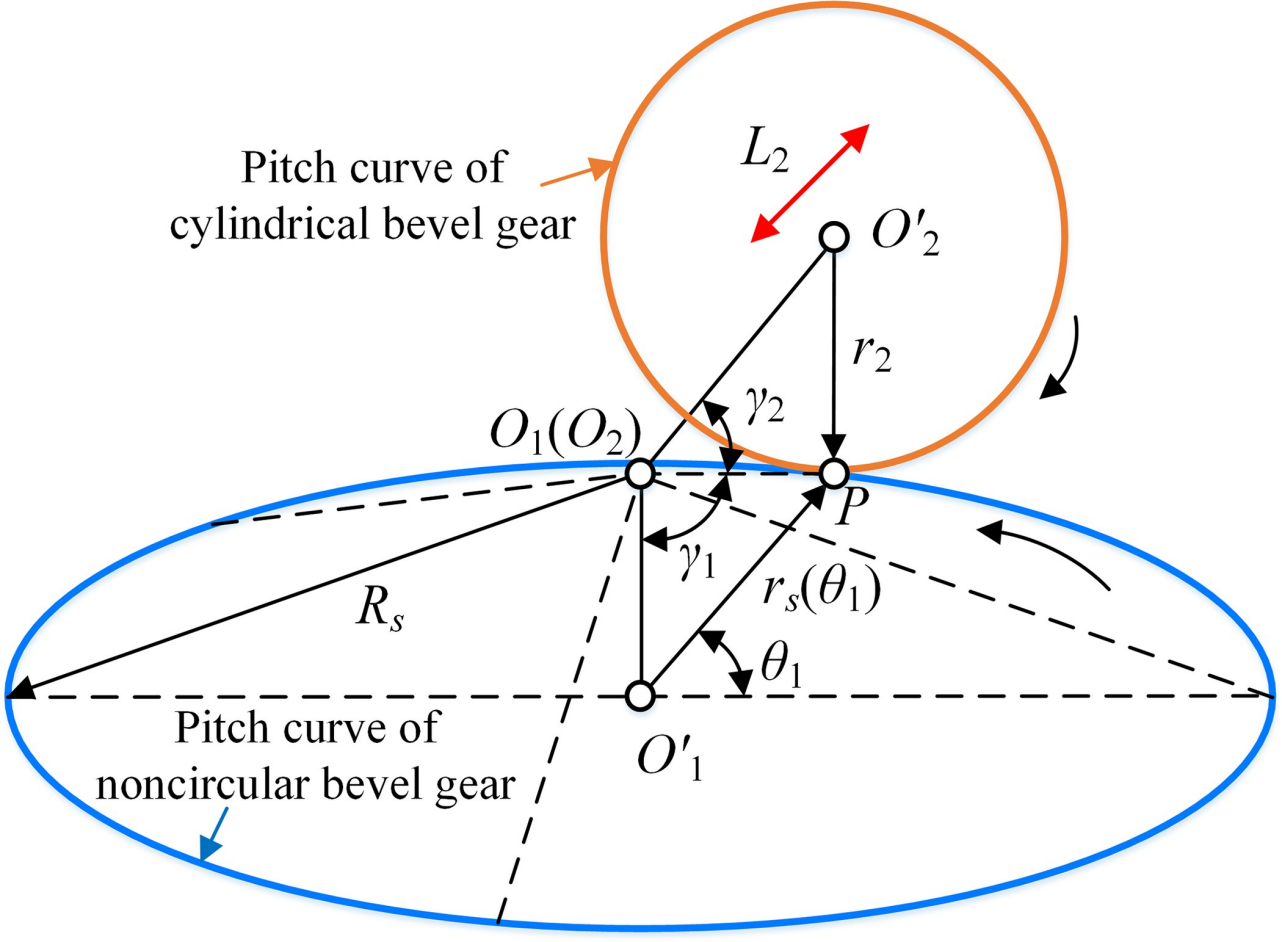
Pitch curve geometry of SNCBG.

SNCBGP are composed of SBG and SNCBG. The compound motion of SBG was realized through the shape of the pitch curve of SNCB, as shown in Fig 7. *R*_*s*_ is the distance between the pitch curve of SNCBG and point *O*_1_. *γ*_1_ and *γ*_2_ are the pitch angles of SNCBG and SBG, respectively. *O*_1_(*O*_2_) is the origin of the corresponding coordinate system. *O*′_1_ and *O*′_2_ are the geometric center of the two pitch curves respectively. P is the meshing point. Assuming the equation of the pitch curve of SNCBG in *S*_1_ is

$$r_{1}(\gamma_{1},\theta_{1})={\left[\begin{array}{cccc} R_{s}\sin\gamma_{1}\cos\theta_{1} & -R_{s}\sin\gamma_{1}\sin\theta_{1} & -R_{s}\cos\gamma_{1} & 1 \end{array}\right]}^{T}$$ (13)

Then the pitch curve of SBG in *S*_2_ can be expressed as

$$r_{2}(\gamma_{2},\theta_{2})=M_{21}r_{1}(\delta_{1},\theta_{1})={\left[\begin{array}{cccc} R_{s}\sin\gamma_{2}\cos\theta_{2} & -R_{s}\sin\gamma_{2}\sin\theta_{2} & R_{s}\cos\gamma_{2}-L_{2}(\theta_{1}) & 1 \end{array}\right]}^{T}$$ (14)

When the pitch curve of SNCBG is a plane curve *r*_*s*_(*θ*_1_), the corresponding parameters can be obtained by

$$\left\{ \begin{array}{l} R_{s}(\theta_{1})=\sqrt{r_{s}^{2}(\theta_{1})+r_{2}^{2}},L_{2}(\theta_{1})=r_{s}(\theta_{1})-r_{s}(0)\\ \tan\gamma_{1}=\frac{r_{s}(\theta_{1})}{r_{2}},\gamma_{2}=\frac{\pi }{2}-\gamma_{1} \end{array}\right.$$ (15)

The pitch curve equations of SNCBG and SBG are

$$\left\{ \begin{array}{l} r_{1}(\gamma_{1},\theta_{1})={\left[\begin{array}{cccc} r_{s}(\theta_{1})\cos\theta_{1} & -r_{s}(\theta_{1})\sin\theta_{1} & -r_{2} & 1 \end{array}\right]}^{T}\\ r_{2}(\gamma_{2},\theta_{2})={\left[\begin{array}{cccc} r_{2}\cos\theta_{2} & -r_{2}\sin\theta_{2} & r_{s}(\theta_{1})-L_{2}(\theta_{1}) & 1 \end{array}\right]}^{T} \end{array}\right.$$ (16)

The two pitch curves are pure rolling, which means the corresponding arc lengths of the two curves are equal, then

$$\int_{0}^{\theta_{1}}\sqrt{r_{s}^{2}(\theta_{1})+{\left[r_{s}^{\prime }(\theta_{1})\right]}^{2}}d\theta =r_{2}\theta_{2}$$ (17)

Where, $r_{s}^{\prime }(\theta_{1})=dr_{s}(\theta_{1})/d\theta_{1}$.

Then the transmission ratio of SNCBGP is

$$i_{21}(\theta_{1})=\frac{d\theta_{2}}{d\theta_{1}}=\frac{\sqrt{r_{s}^{2}(\theta_{1})+{\left[r_{s}^{\prime }(\theta_{1})\right]}^{2}}}{r_{2}}$$ (18)

**Fig 8:**
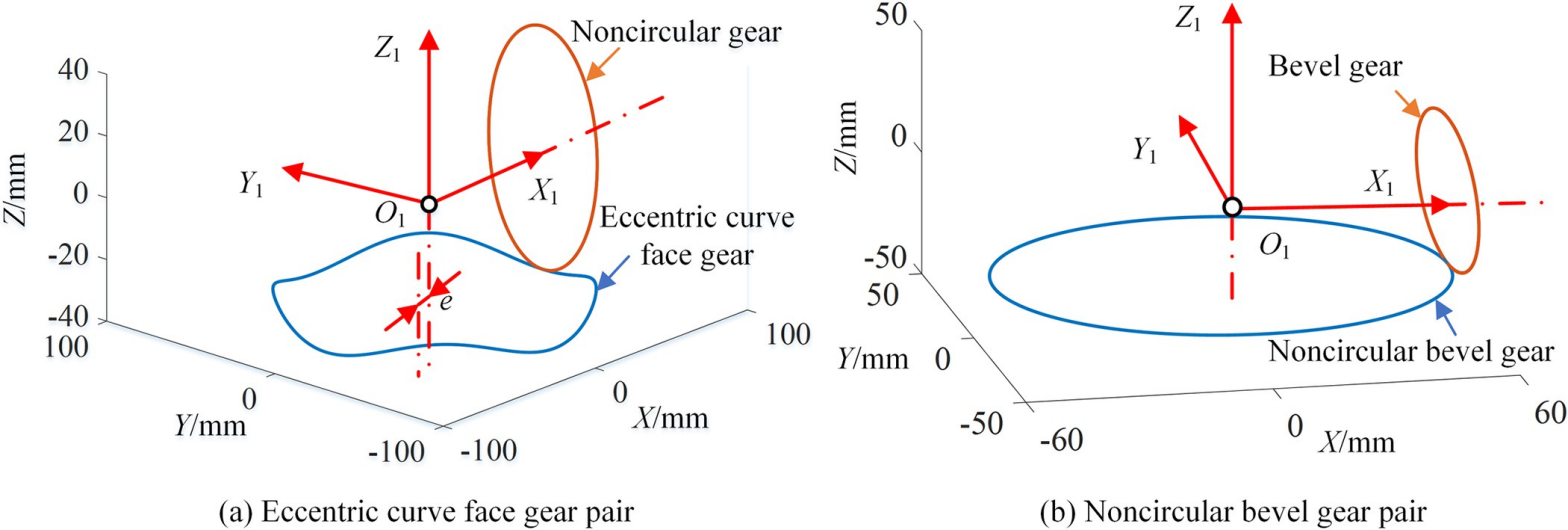
Pitch curves of type II.

For ECFGP and SNCBGP, different compound transmission can be realized by changing *r*_2_(*θ*_2_) and *r*_*s*_(*θ*_1_) respectively. When both *r*_2_(*θ*_2_) and *r*_*s*_(*θ*_1_) adopt the equation in Eq (19), the pitch curves can be obtained by taking the parameters in Table 3, as shown in Fig 8. *n*_1_ is the order of ECFG or SNCBG, and *n*_2_ is the order of NCG or SBG.


$$r(\theta )=\frac{a(1-k^{2})}{1-k\cos n\theta }$$ (19)

Where, *a*, *k* and *n* are the radius of the major axis, eccentricity and order of the ellipse, respectively.

**Table 3:** Pitch curve parameters of type II.

Gear pair	*a*	*k*	*n*_1_	*n*_2_	*R*_*c*_	*e*	*r*_2_
ECFGP	35.8mm	0.1	4	2	71.3mm	5mm	/
NCBGP	50.4mm		2	/	/	/	30.6mm

As envisaged, in Fig 8(A), the pitch curve of NCG is a planar ellipse and the pitch curve of ECFG is a cylindrical curve. In Fig 8(B), the pitch curve of SNCBG is a planar ellipse and the pitch curve of SBG is a circle. Specially, when *L*_2_ is 0, the transmission can be changed into a fixed type by selecting appropriate parameters.

### Instant screw axis of SNCG compound motion

**Fig 9:**
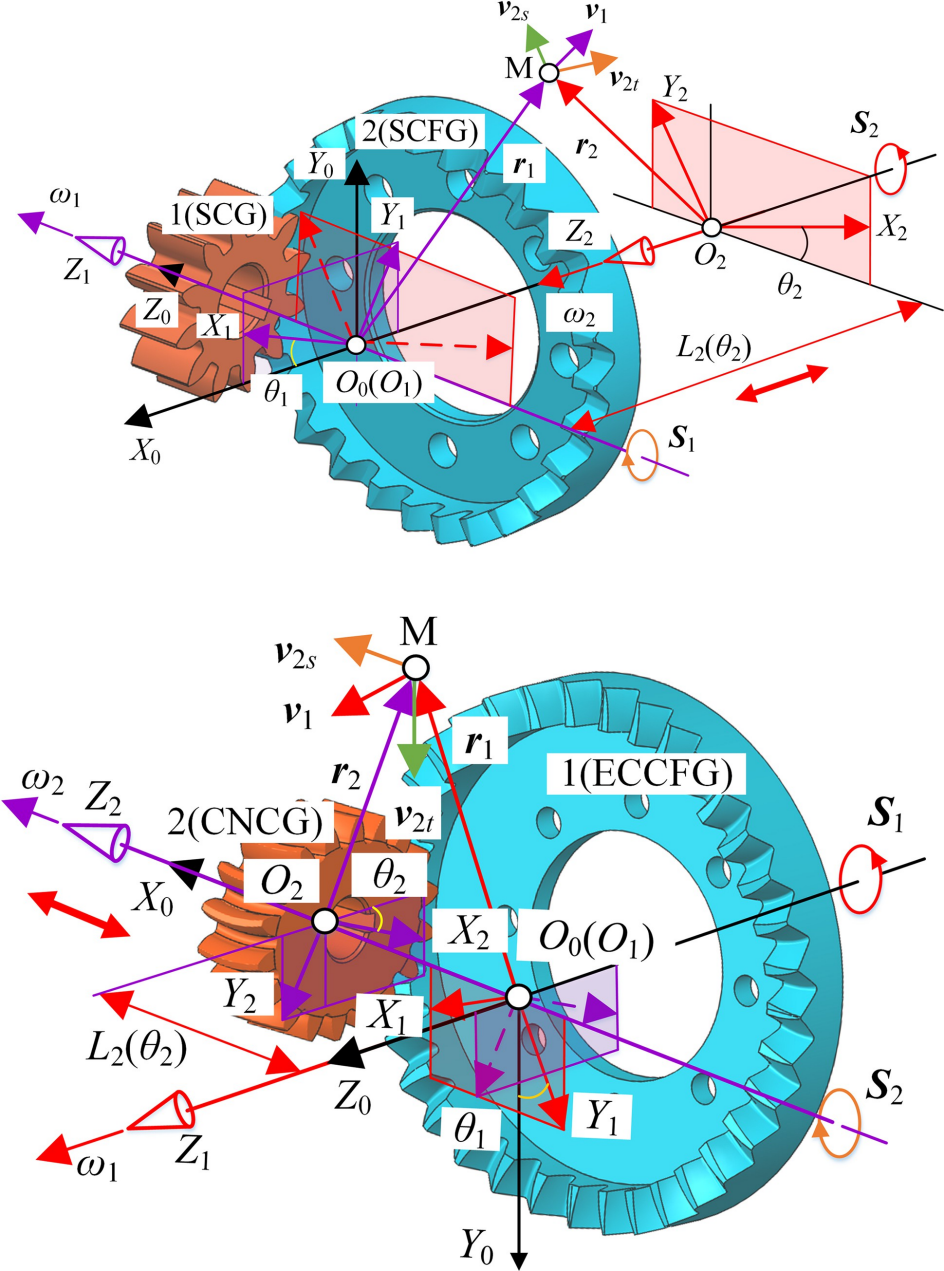
Screw analysis of compound transmission. (a) Type I, (b) Type II.

As shown in Fig 9, the motion screw of each type was analyzed, and the corresponding parameters have the same meaning as before. M is the meshing point. ***r***_1_ and ***r***_2_ are the position vectors of point M in *S*_1_ and *S*_2_ respectively. ***v***_1_ is the velocity of point M on gear 1. ***v***_2*s*_ and ***v***_2*t*_ are the axial velocity and tangential velocity of point M on gear 2 respectively.

For both types, the screw analysis method is the same. In *S*_0_, the screw of gear 1 is

$$S_{1}={\left(0,0,\omega_{1},0,0,0\right)}^{T}$$ (20)

In *S*_*f*_, the screw $S_{2}^{\left(f\right)}$ of gear 2 relative to gear 1 is

$$S_{2}^{\left(f\right)}=({\vec{C}}_{2}^{\left(f\right)};{\vec{\zeta }}_{2}^{\left(f\right)})={\left(0,0,\omega_{2}(\theta_{2}),0,0,v_{2s}(\theta_{2})\right)}^{T}$$ (21)

Where, ${\vec{C}}_{2}^{\left(f\right)}$ and ${\vec{\zeta }}_{2}^{\left(f\right)}$ are the primary part (main vector) and secondary part of $S_{2}^{\left(f\right)}$ respectively, and $v_{2s}(\theta_{2})=\omega_{2}(\theta_{2})h_{2}(\theta_{2})$.

Then in *S*_0_, the screw $S_{2}^{\left(0\right)}$ of gear 2 relative to gear 1 is

$$S_{2}^{\left(0\right)}=({\vec{C}}_{2}^{\left(0\right)};{\vec{\zeta }}_{2}^{\left(0\right)})=(L_{0f}{\vec{C}}_{2}^{\left(f\right)};L_{0f}({\vec{\zeta }}_{2}^{\left(f\right)}+{\left(0,E(\theta_{1}),0\right)}^{T}\times {\vec{C}}_{2}^{\left(f\right)}))={\left(\omega_{2}(\theta_{2}),0,0;v_{2s}(\theta_{2}),0,0\right)}^{T}$$ (22)

Where, ${\vec{C}}_{2}^{\left(f\right)}$ and ${\vec{\zeta }}_{2}^{\left(f\right)}$ are the primary part and secondary part of $S_{2}^{\left(0\right)}$ respectively. ***L***_0*f*_ is a submatrix consisting of the first three rows and three columns of ***M***_0*f*_, similarly below.

Moreover, in *S*_0_, the screw $S_{f}^{\left(0\right)}$ of the axis of gear 2 is **0**. Then the total screw of gear 2 in *S*_0_ is

$$S_{2}=S_{2}^{\left(0\right)}+S_{f}^{\left(0\right)}$$ (23)

Instant screw axis refers to the axis where the relative screw of a pair of gears is located. Here, the relative screw ***S***_*is*_ of gear 2 relative to gear 1 in *S*_0_ is

$$S_{is}=({\vec{C}}_{is};{\vec{\zeta }}_{is})=S_{2}-S_{1}={\left(\omega_{2}(\theta_{2}),0,-\omega_{1};v_{2s}(\theta_{2}),0,0\right)}^{T}$$ (24)

Where, ${\vec{C}}_{is}^{\left(f\right)}$ and ${\vec{\zeta }}_{is}^{\left(f\right)}$ are the primary part and secondary part of ***S***_*is*_, respectively.

The direction vector ***s***_*is*_, the pitch *h*_*is*_ and the normal vector ***r***_*isn*_ passing through the origin *O*_0_ of the instant screw axis can be obtained by Eq (25).


$$\left\{ \begin{array}{l} s_{is}=\frac{{\vec{C}}_{is}}{\left|{\vec{C}}_{is}\right|}={\left(\frac{\omega_{2}(\theta_{2})}{\sqrt{\omega_{1}^{2}+\omega_{2}^{2}(\theta_{2})}},0,\frac{-\omega_{1}}{\sqrt{\omega_{1}^{2}+\omega_{2}^{2}(\theta_{2})}}\right)}^{T}\\ h_{is}=\frac{{\vec{C}}_{is}\cdot {\vec{\zeta }}_{is}}{{\vec{C}}_{is}\cdot {\vec{C}}_{is}}=\frac{\omega_{2}(\theta_{2})v_{2s}(\theta_{2})}{\omega_{1}^{2}+\omega_{2}^{2}(\theta_{2})}\\ r_{isn}=\frac{{\vec{C}}_{is}\times {\vec{\zeta }}_{is}}{{\vec{C}}_{is}\cdot {\vec{C}}_{is}}={\left(0,\frac{-\omega_{1}v_{2s}(\theta_{2})}{\omega_{1}^{2}+\omega_{2}^{2}(\theta_{2})},0\right)}^{T} \end{array}\right.$$ (25)

Then the linear vector equation of the instant screw axis can be expressed as

$${\vec{l}}_{is}(\lambda )=r_{isn}+\lambda {\vec{s}}_{is}={\left(\frac{\lambda \omega_{2}(\theta_{2})}{\sqrt{\omega_{1}^{2}+\omega_{2}^{2}(\theta_{2})}},\frac{-\omega_{1}v_{2s}(\theta_{2})}{\omega_{1}^{2}+\omega_{2}^{2}(\theta_{2})},\frac{-\lambda \omega_{1}}{\sqrt{\omega_{1}^{2}+\omega_{2}^{2}(\theta_{2})}}\right)}^{T}$$ (26)

Where, *λ* is the distance from any point on the line to the position vector ***r***_*isn*_, positive in the direction of ***s***_*is*_.

**Fig 10:**
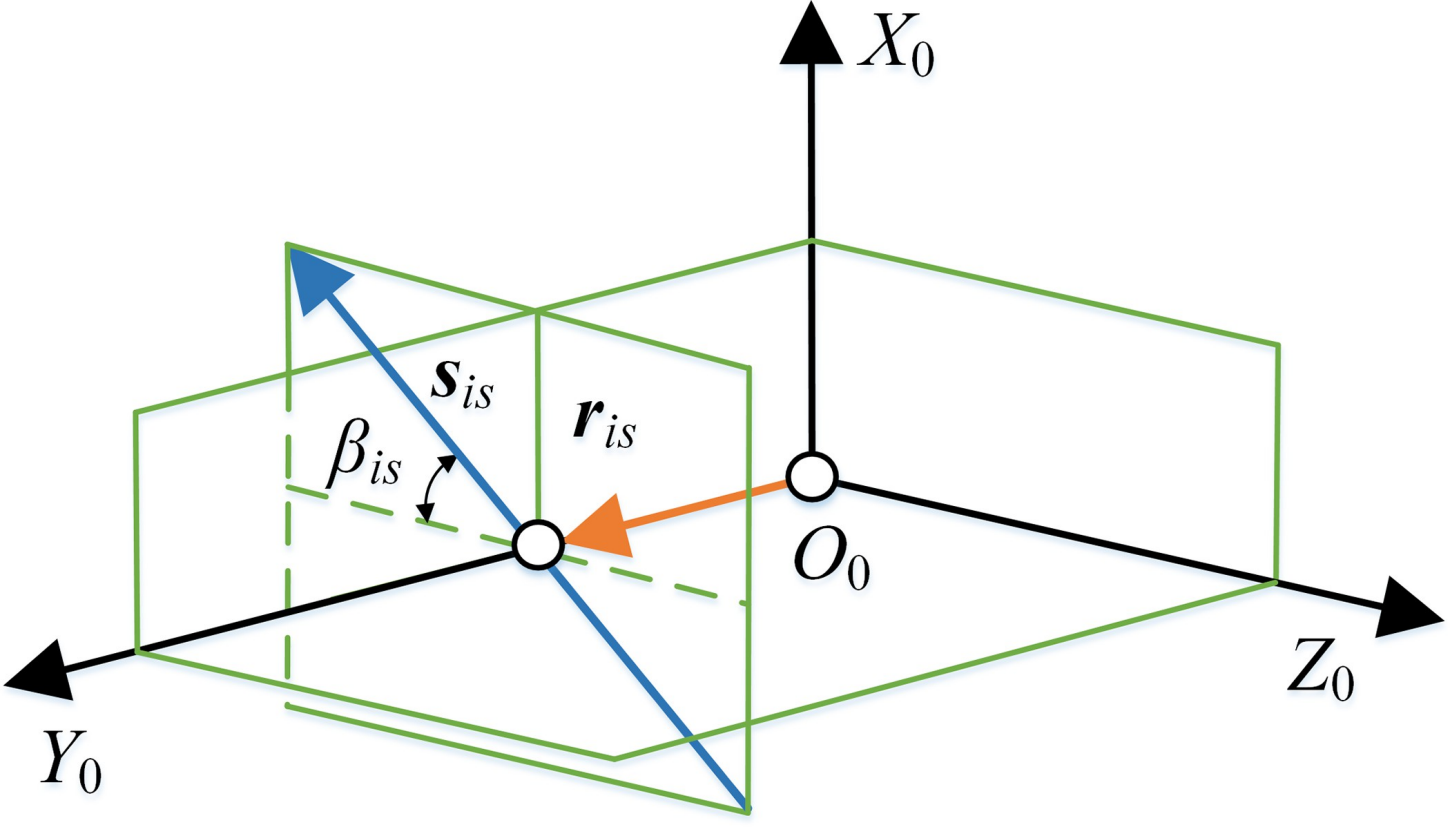
Spatial pose of instant screw axis.

For a general spatial gear trasnmission, the instant screw axis is a straight line in space, and its spatial pose is related to the direction vector ***s***_*is*_ and the normal vector ***r***_*isn*_. During the gear transmission, the instant screw axis moves in a spiral, that is, rotates around the axis and moves along the axial direction simultaneously. The instant screw axis of SNCG compound transmission is a special case of that of the general spatial gear trasnmission. According to Eq (25), The direction vector ***s***_*is*_ is parallel to *X*_0_*O*_0_*Z*_0_, and the normal vector ***r***_*isn*_ recombines with *O*_0_*Y*_0_. The spatial pose of the instant screw axis was shown in Fig10. The instant screw axis is a straight line that vertically intersects with axis *O*_0_*Y*_0_ and forms an included angle *β*_*is*_ with plane *Y*_0_*O*_0_*Z*_0_. *β*_*is*_ = arctan(*ω*_2_/*ω*_1_).

### Axodes of SNCG compound transmission

Axode is an important basis for gear design and manufacturing and it can be obtained by rotating the instant screw axis around the respective rotation axis of each gear. Base on the above analysis, the axodes of SNCG can be obtained by

$$\left\{ \begin{array}{l} A_{1}(\theta_{1},\lambda )=M_{10}{\vec{L}}_{is}(\lambda )={\left(\frac{\lambda i_{21}\cos\theta_{1}}{\sqrt{1+i_{21}^{2}}}-\frac{h_{2}i_{21}\sin\theta_{1}}{1+i_{21}^{2}},-\frac{\lambda i_{21}\sin\theta_{1}}{\sqrt{1+i_{21}^{2}}}-\frac{h_{2}i_{21}\cos\theta_{1}}{1+i_{21}^{2}},-\frac{\lambda }{\sqrt{1+i_{21}^{2}}},1\right)}^{T}\\ A_{2}(\theta_{2},\lambda )=M_{20}{\vec{L}}_{is}(\lambda )={\left(\frac{\lambda \cos\theta_{2}}{\sqrt{1+i_{21}^{2}}}-\frac{h_{2}i_{21}\sin\theta_{2}}{1+i_{21}^{2}},-\frac{\lambda \sin\theta_{2}}{\sqrt{1+i_{21}^{2}}}-\frac{h_{2}i_{21}\cos\theta_{2}}{1+i_{21}^{2}},\frac{\lambda i_{21}}{\sqrt{1+i_{21}^{2}}}-L_{2}(\theta_{2}),1\right)}^{T} \end{array}\right.$$ (27)

Where, ${\vec{L}}_{\mathrm{is}}(\lambda )$ a homogeneous coordinate formed by adding a row element 1 to ${\vec{l}}_{\mathrm{is}}(\lambda )$, which is used for coordinate transformation. The variation range of *θ*_1_ is [0, 2π] and the variation range of *λ* is [–∞, +∞].

#### Axodes of type I

**Fig 11:**
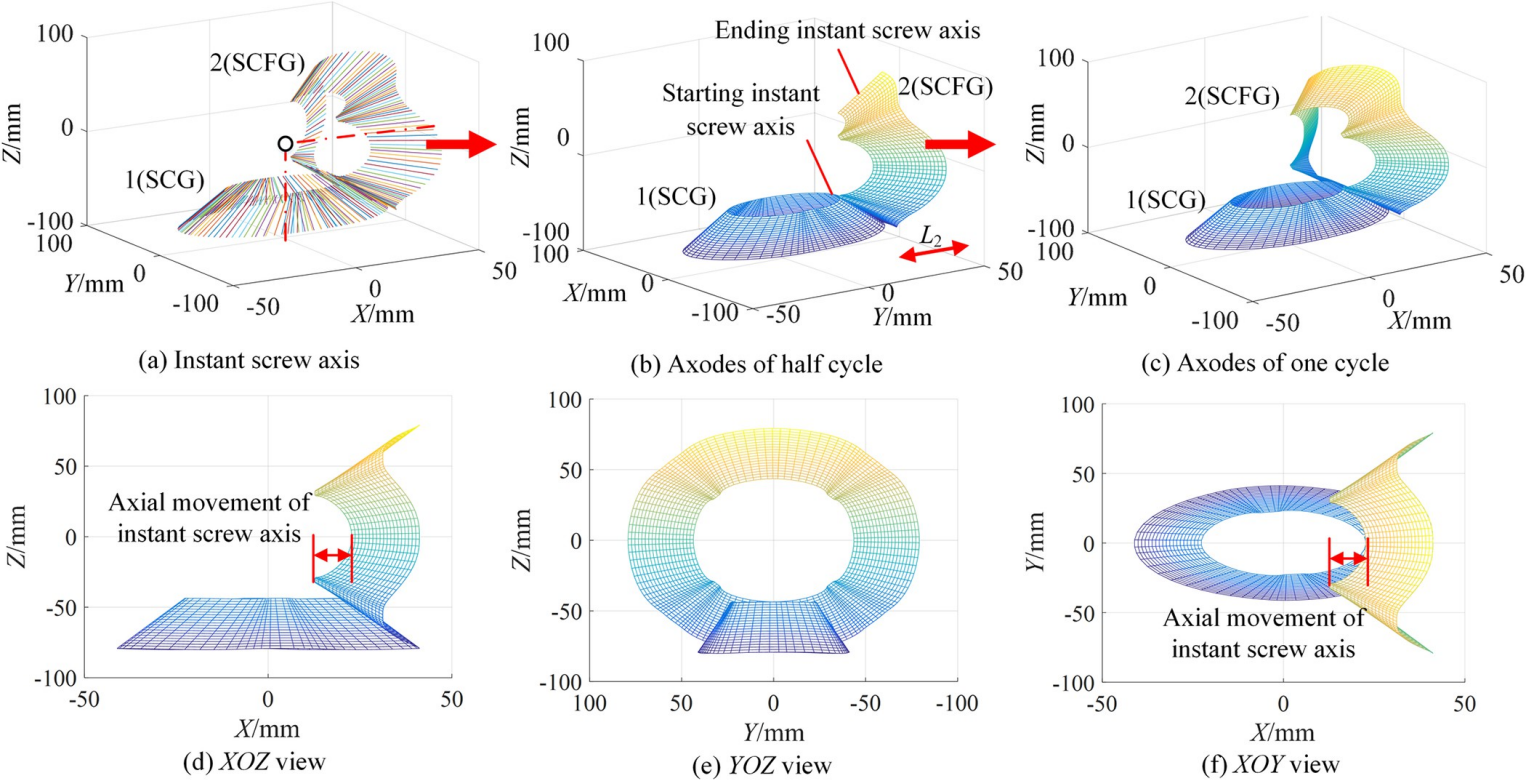
Generation process of axodes of type I.

Taking the transmission ratio and axial displacement of type I as an example, according to Eq (27), select the appropriate λ (around *R*_0_), and we can obtain the generation process of axodes, as shown in Fig 11.

Define that one revolution of SCFG is one cycle. Fig 11(A) shows the rotation process of instant screw axis in one cycle. Fig 11(B) and 11(C) are the axodes of the two gears in a half cycle and a cycle respectively. Fig 11(D)–11(F) are the projections of Fig 11(C) on the corresponding plane. The axodes of SCG and SCFG are nonlinear local helical surfaces. Notably, since *i*_21_ and *L*_2_ are periodic variables, the pitch of ***S***_*is*_ is not zero, that is, the instant screw axis rotates around the axis and moves periodically along the axial direction, so the two axodes exhibit helicity and periodicity.

**Fig 12:**
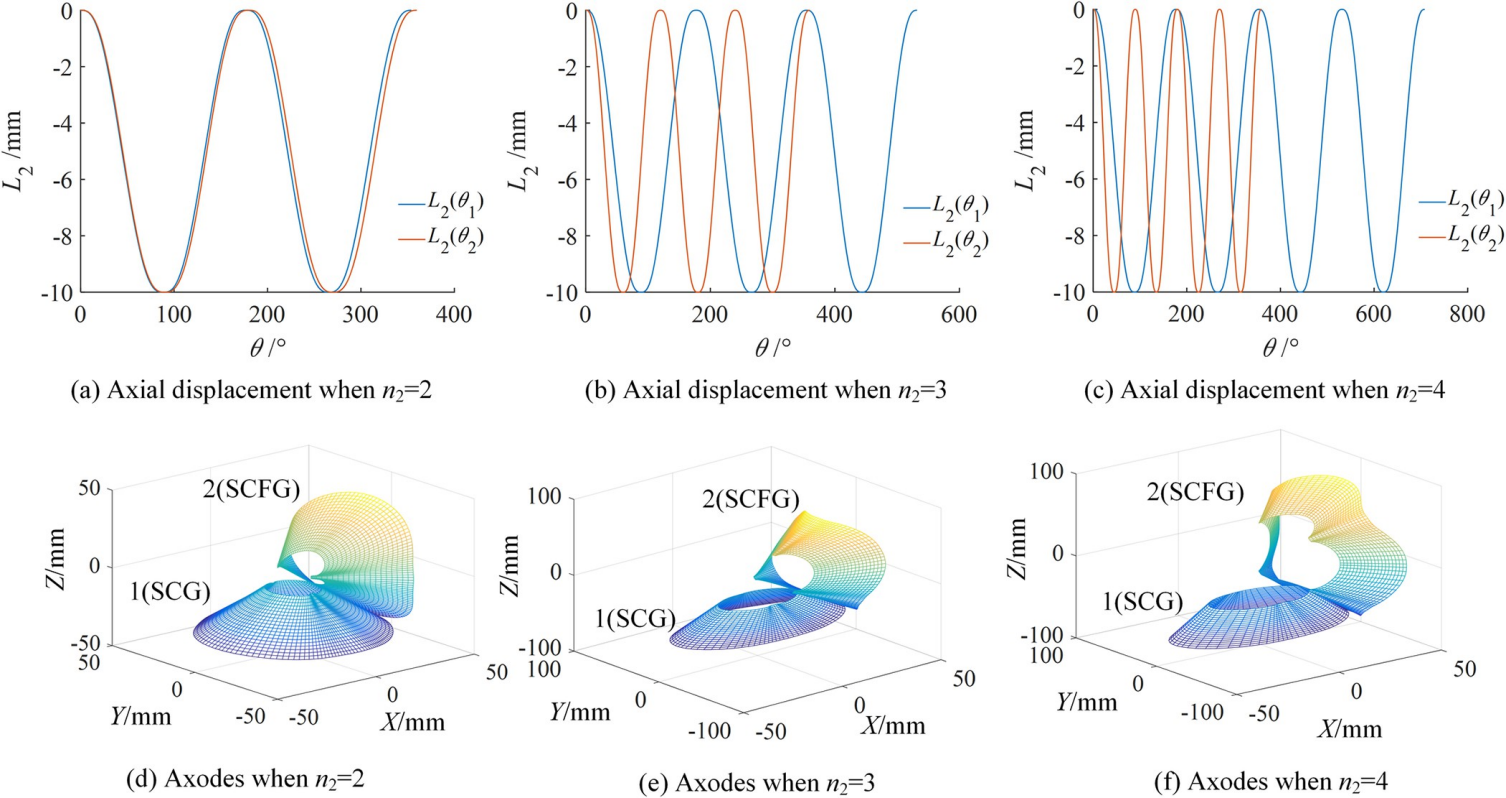
Variation laws of axial displacement and axodes of type I with different *n*_2_.

The order of SCFG has a great influence on the compound transmission. When *n*_2_ = 2,3,4, the variation laws of axial displacement and axodes were obtained, as shown in Fig 12. Fig 12(A)–12(C) show the variation law of *L*_2_ with different *n*_2_. With the increase of *n*_2_, the amplitudes of *L*_2_(*θ*_1_) and *L*_2_(*θ*_2_) remain unchanged, but the numbers of cycles increase. Moreover, the range of *θ*_2_ is 0~360°, which remains unchanged. However, the range of *θ*_1_ gradually increases, and its maximum value is related to *n*_2_. Fig 12(D)–12(F) are the corresponding axodes respectively. Because the instant screw axis rotates around the axis and moves periodically along the axial direction, all axodes were called nonlinear local helical surfaces, exhibiting helicity and periodicity, and the specific shape is related to the transmission ratio and axial displacement.

#### Axodes of type II

**Fig 13:**
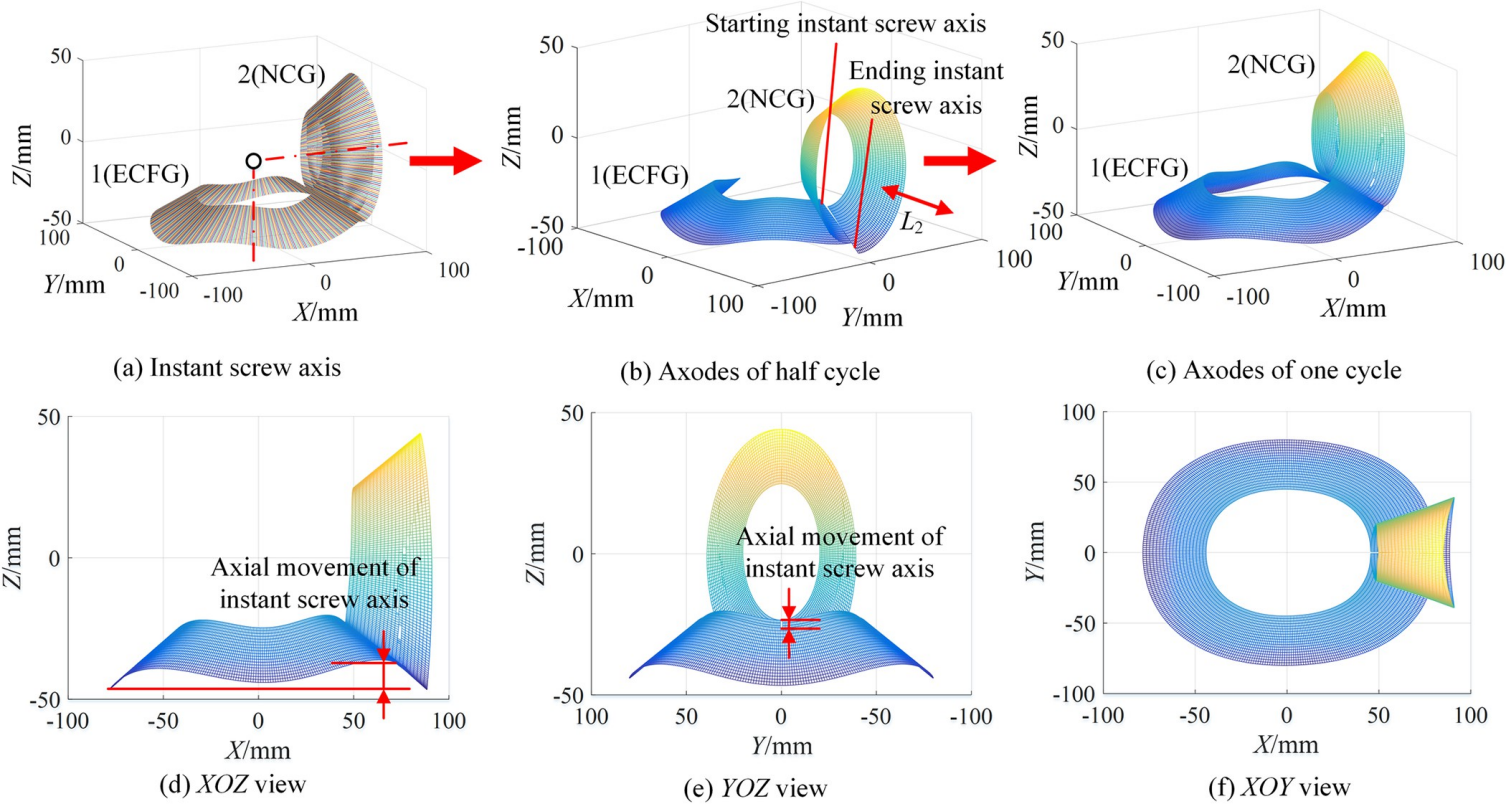
Generation process of axodes of type II.

For type II, the axodes of ECFGP and SNCBGP are similar. In order to reduce the length, ECFGP was analyzed as an example. Taking the transmission ratio and axial displacement of type II as an example, according to Eq (27), select the appropriate λ (around *R*_*c*_), and the generations process of axodes can be obtained as shown in Fig 13. Define that one revolution of ECFG is one cycle. Fig 13(A) shows the rotation process of the instant screw axis. Fig 13(B) and 13(C) are the axodes of the two gears in a half cycle and a cycle, respectively. Fig 13(D)–13(F) are the projections of Fig 13(C) on the corresponding plane. The axodes of NCG and ECFG are nonlinear local helical surfaces. Similarly, since *i*_21_ and *L*_2_ are periodic variables, the two axodes exhibit helicity and periodicity.

**Fig 14:**
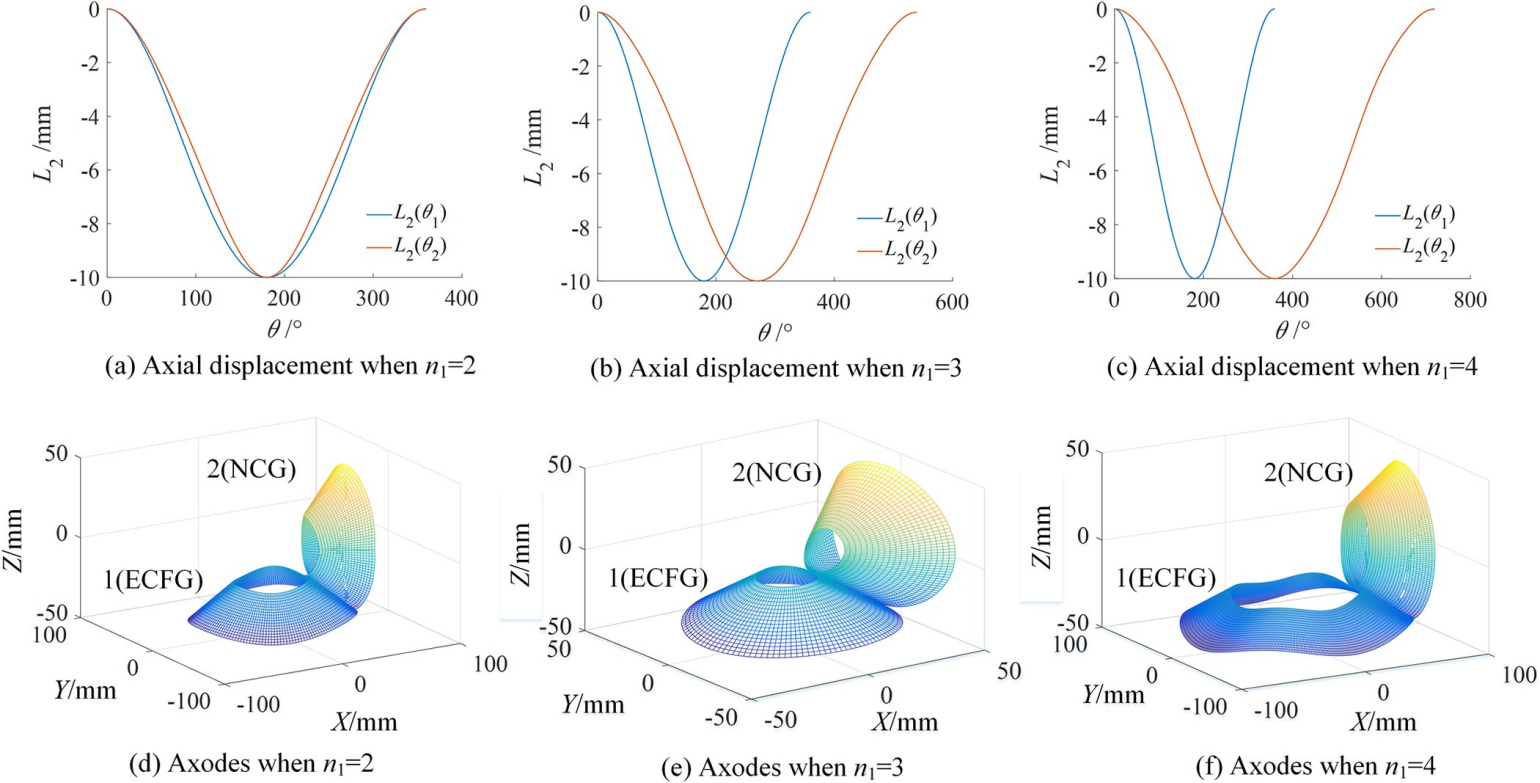
Variation laws of axial displacement and axodes of type II with different *n*_1_.

The order of ECFG has a great influence on the compound transmission. When *n*_1_ = 2,3,4 and *n*_2_ = 2, the variation laws of axial displacement and axodes were obtained, as shown in Fig 14. Fig 14(A)–14(C) show the variation law of *L*_2_ with different *n*_1_. With the increase of *n*_1_, *L*_2_(*θ*_1_) remain unchanged and the amplitude of *L*_2_(*θ*_2_) does not change. The range of *θ*_2_ gradually increases and the maximum of *θ*_2_ is 2π*n*_1_/*n*_2_. Fig 14(D)–14(F) are the corresponding axodes respectively. Similarly, because the instant screw axis rotates around the axis and moves periodically along the axial direction, all axodes were called nonlinear local helical surfaces, exhibiting helicity and periodicity, and the specific shape of each axode is related to the transmission ratio and axial displacement.

In summary, the axodes of SNCG compound transmission are nonlinear local helical surfaces and the specific shape is related to the spatial position, transmission ratio and axial displacement of each gear pair.

### Pitch surface of SNCG compound transmission

Pitch surface is the reference surface of gear transmission. For SNCG compound transmission, the pitch of instant screw axis is not zero, that is, there is a relative sliding along the instant screw axis, which limits the tangential direction of the tooth profile. Combined with the relative velocity and corresponding normal vector at the reference point, the pitch surfaces of the gear pairs can be obtained. For SNCG compound transmission with known transmission ratio, its instant screw axis is unique. Take the reference point P on the instant screw axis and assume its position parameter is *λ*, then the position vector of point P is

$$r_{pl}(\theta )={\vec{l}}_{is}(\lambda_{p},i_{21}(\theta ))={\left(\frac{\lambda_{p}i_{21}(\theta )}{\sqrt{1+i_{21}^{2}(\theta )}},\frac{-h_{2}i_{21}(\theta )}{1+i_{21}^{2}(\theta )},\frac{-\lambda_{p}}{\sqrt{1+i_{21}^{2}(\theta )}}\right)}^{T}$$ (28)

Where, *θ* is *θ*_1_ or *θ*_2_.

**Fig 15:**
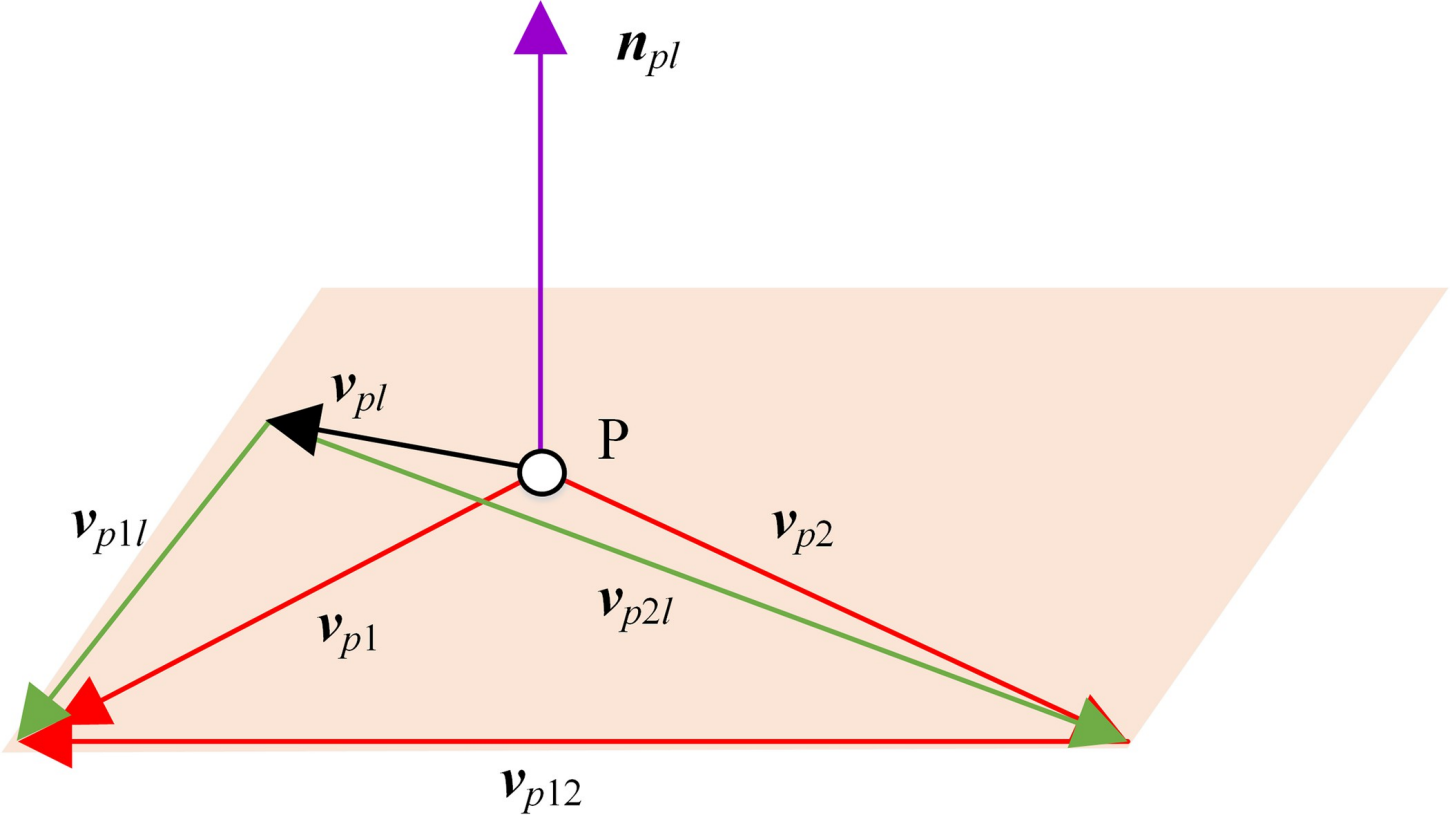
Pitch surface generation.

Then we can discuss the velocity of point P, as shown in Fig 15. At point P, the velocity ***v***_*pl*_ induced by the instant screw axis, the velocity ***v***_*p*1_ of gear 1, and the velocity ***v***_*p*2_ of gear 2 are respectively

$$\left\{ \begin{array}{l} v_{pl}(\theta )=dr_{pl}(\theta )/d(\theta )\\ v_{p1}(\theta )={\vec{C}}_{1}\times r_{pl}(\theta )\\ v_{p2}(\theta )={\vec{C}}_{2}^{\left(0\right)}\times r_{pl}(\theta )+h_{2}{\vec{C}}_{2}^{\left(0\right)} \end{array}\right.$$ (29)

At point P, the velocities of gear 1 and gear 2 relative to the instant screw axis are ***v***_*p*1*l*_ and ***v***_*p*2*l*_ respectively.


$$\left\{ \begin{array}{l} v_{p1l}(\theta )=v_{p1}(\theta )-v_{pl}(\theta )\\ v_{p2l}(\theta )=v_{p2}(\theta )-v_{pl}(\theta ) \end{array}\right.$$ (30)

The normal vector of pitch surface at point P is

$$n_{pl}(\theta )=v_{p1l}(\theta )\times v_{p2l}(\theta )$$ (31)

A reference point can only generate a curve, which need to be extended into a space surface. Considering that the extended surfaces need to be tangent, that is, to ensure that the position of the tangent plane and the normal vector are unchanged.

Taking ***v***_*p*1*l*_ and ***v***_*p*2*l*_ as the first tangent vectors of the pitch surfaces of gear 1 and gear 2 at point P respectively, the second tangent vectors of the pitch surfaces at point P are

$$\left\{ \begin{array}{l} t_{p1}(\theta )=n_{pl}(\theta )\times v_{p1l}(\theta )\\ t_{p2}(\theta )=n_{pl}(\theta )\times v_{p2l}(\theta ) \end{array}\right.$$ (32)

By giving the position parameter *t*_*p*_, point P can be extended to two straight lines respectively, as follows

$$\left\{ \begin{array}{l} {\vec{l}}_{p1}(\theta ,t_{p})=r_{pl}(\theta )+t_{p}{\bar{t}}_{p1}(\theta )\\ {\vec{l}}_{p2}(\theta ,t_{p})=r_{pl}(\theta )+t_{p}{\bar{t}}_{p2}(\theta ) \end{array}\right.$$ (33)

Where, ${\bar{t}}_{p1}$ and ${\bar{t}}_{p2}$ are the unit vectors of ***t***_*p*1_ and ***t***_*p*2_ respectively.

Thus, the conjugate pitch surfaces of gear 1 and gear 2 can be obtained as

$$\left\{ \begin{array}{l} A_{P1}(\theta ,t_{p})=M_{10}{\vec{l}}_{p1}(\theta ,t_{p})\\ A_{P2}(\theta ,t_{p})=M_{20}{\vec{l}}_{p2}(\theta ,t_{p}) \end{array}\right.$$ (34)

### Normal equidistant surface of pitch surface

The surfaces enveloped by the crest and root of generator are the normal equidistant surfaces of the pitch surfaces. Generally, the generating lines of the pitch surfaces of gear 1 and gear 2 can be represented by ${\vec{l}}_{p1}(\theta ,t_{p})$ and ${\vec{l}}_{p2}(\theta ,t_{p})$.

The common normal vector of the two pitch surfaces at the meshing point is ***n***_*pl*_, then the generating lines of normal equidistant surfaces of pitch surfaces can be expressed as

$$\left\{ \begin{array}{l} {\vec{l}}_{n1}(\theta ,t_{p},h_{n})={\vec{l}}_{p1}(\theta ,t_{p})+h_{n}n_{pl}\\ {\vec{l}}_{n2}(\theta ,t_{p},h_{n})={\vec{l}}_{p2}(\theta ,t_{p})+h_{n}n_{pl} \end{array}\right.$$ (35)

Where, *h*_*n*_ is the normal distance, and ${\bar{n}}_{\mathrm{pl}}$ is the unit vectors of ***n***_*pl*_.

Finally, the normal equidistant surfaces of the pitch surfaces can be obtained as

$$\left\{ \begin{array}{l} A_{Pn1}(\theta ,t_{p})=M_{10}{\vec{l}}_{n1}(\theta ,t_{p},h_{n})\\ A_{Pn2}(\theta ,t_{p})=M_{20}{\vec{l}}_{n2}(\theta ,t_{p},h_{n}) \end{array}\right.$$ (36)

When *h*_*n*_ is the addendum *h*_*a*_, Eq (36) represents the tip surface. When *h*_*n*_ is the dedendum–*h*_*f*_, Eq (36) represents the root surface. The tip surface plays an important role in the blank design of gear machining.

## Tooth surface geometry of SNCG

From the previous analysis, it can be known that the pitch surface of SNCG is based on the relative velocity relationship, the pitch surfaces of point meshing are instantaneous tangent and their normal directions at the meshing point are consistent. According to the meshing theory, for a gear pair, as long as the direction of the relative velocity is perpendicular to the normal direction of the tooth profile, the gear transmission can be correct. At this moment, if the generator is constructed on the common tangent surface of the pitch surfaces of two gears, so that the tangent direction of the tooth profile of generator is consistent with the direction of the relative speed of the two gears, a pair of conjugate tooth profiles can theoretically be produced. In fact, the two tooth profiles are generally point meshing. If and only if the axodes are selected as the pitch surfaces, and the velocities of generator relative to the two gears are along the direction of the instant screw axis everywhere, the theoretical conjugate tooth profiles of line meshing can be generated. According to the screw analysis of compound transmission, tooth surface and motion process of generator, the equation of the tooth surface of SNCG can be obtained.

### Tooth surface geometry of generator

#### Rack generator

**Fig 16:**
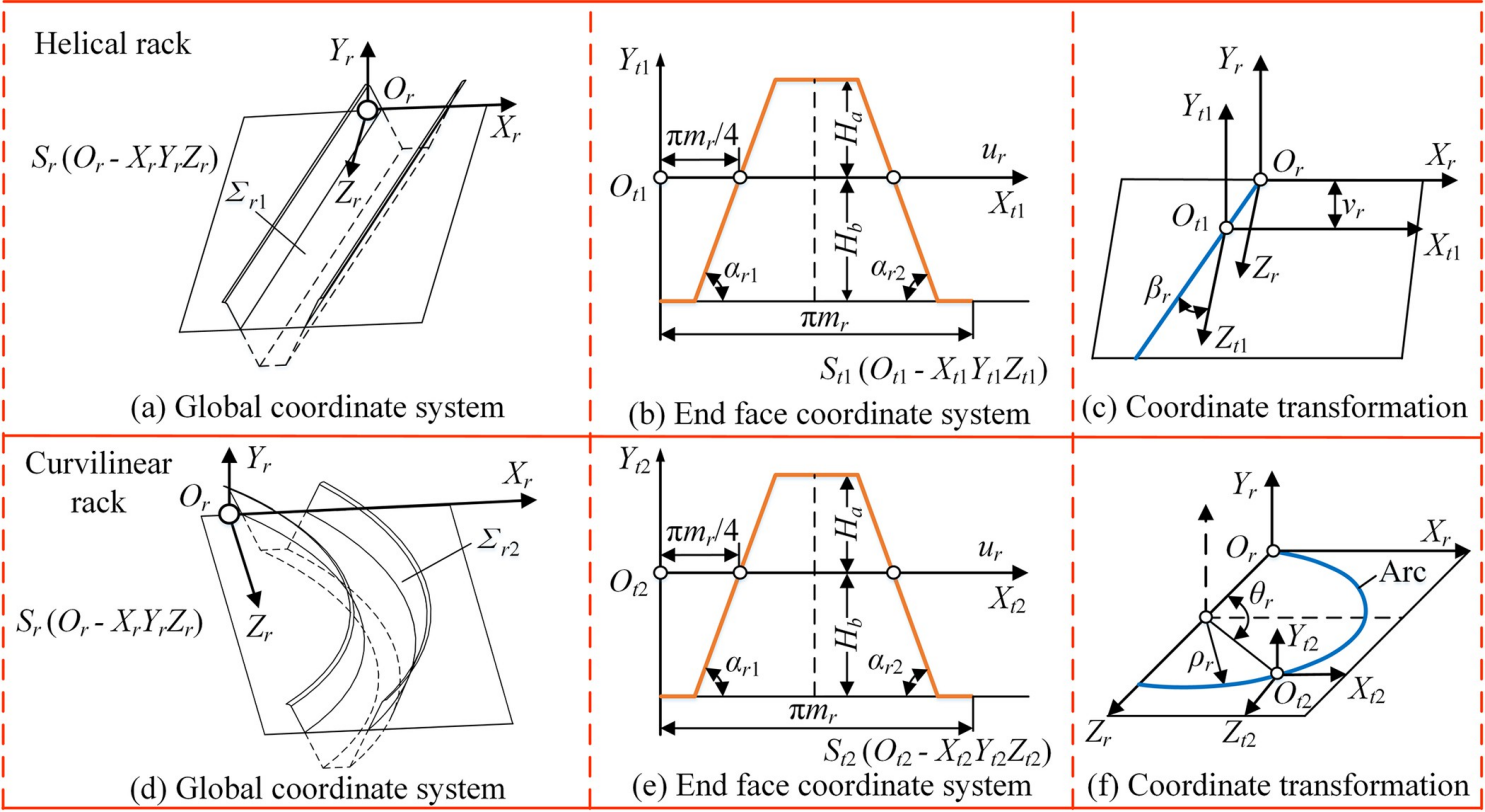
The coordinate system of the tooth surface of rack generator.

The tooth surface *Σ*_*r*_ of rack generator includes straight tooth, helical tooth, curvilinear tooth and herringbone tooth. Fig 16 is the tooth surface coordinate system when the tooth profile is helical tooth or curvilinear tooth. The global coordinate systems of the helical and curvilinear rack are both expressed as *S*_*r*_ (*O*_*r*_*−X*_*r*_*Y*_*r*_*Z*_*r*_), abbreviated as *S*_*r*_, as shown in Fig 16(A) and 16(D). In Fig 16(B), *S*_*t*1_ (*O*_*t*1_ *–X*_*t*1_*Y*_*t*1_*Z*_*t*1_), abbreviated as *S*_*t*1_, is the end face coordinate system of helical rack. *O*_*t*1_*X*_*t*1_ coincides with the pitch line of the rack and *O*_*t*1_*Y*_*t*1_ coincides with the cogging symmetry line. *H*_*a*_ and *H*_*b*_ are the addendum and dedendum respectively. *m*_*r*_ is the module of the rack. *α*_*r*1_ and *α*_*r*2_ are the left and right tooth profile angles respectively. In Fig 16(E), *S*_*t*2_ (*O*_*t*2_ *–X*_*t*2_*Y*_*t*2_*Z*_*t*2_), abbreviated as *S*_*t*2_, is the end face coordinate system of curvilinear rack. The tooth profiles of curvilinear rack and helical rack in the direction of tooth width are the same. Fig 16(C) and 16(F) show the transformation process from *S*_*t*1_ and *S*_*t*2_ to *S*_*r*_ respectively. *u*_*r*_ and *v*_*r*_ are the tooth profile parameters and tooth width parameters of the rack respectively. *β*_*r*_ is the helical angle of the helical rack. *θ*_*r*_ and *ρ*_*r*_ are the arc angle and arc radius of the curvilinear rack respectively.

The rack adopts an involute tooth profile with the bilateral pressure angle, which can be expressed as

$$\left\{ \begin{array}{l} r_{t1}(u_{r})=r_{t2}(u_{r})={\left[\begin{array}{cccc} u_{r} & f(u_{r}) & 0 & 1 \end{array}\right]}^{T}\\ f(u_{r})=\left\{ \begin{array}{l} -H_{b},u_{r}\in [0,\frac{\pi m_{r}}{4}-H_{b}\tan\alpha_{r1})\\ \frac{1}{\tan\alpha_{r1}}(u_{r}-\frac{\pi m_{r}}{4}),u_{r}\in [\frac{\pi m_{r}}{4}-H_{b}\tan\alpha_{r1},\frac{\pi m_{r}}{4}+H_{a}\tan\alpha_{r1})\\ H_{a},u_{r}\in [\frac{\pi m_{r}}{4}+H_{a}\tan\alpha_{r1},\frac{3\pi m_{r}}{4}-H_{a}\tan\alpha_{r2})\\ \frac{1}{\tan\alpha_{r2}}(\frac{3\pi m_{r}}{4}-u_{r}),u_{r}\in [\frac{3\pi m_{r}}{4}-H_{a}\tan\alpha_{r2},\frac{3\pi m_{r}}{4}+H_{b}\tan\alpha_{r2})\\ -H_{b},u_{r}\in [\frac{3\pi m_{r}}{4}+H_{b}\tan\alpha_{r2},\pi m_{r}] \end{array}\right. \end{array}\right.$$ (37)

The transformation matrices from *S*_*t*1_ and *S*_*t*2_ to *S*_*r*_ are

$$M_{rt1}(v_{r})=[\begin{array}{cccc} 1 & 0 & 0 & -v_{r}\tan\beta_{r}\\ 0 & 1 & 0 & 0\\ 0 & 0 & 1 & v_{r}\\ 0 & 0 & 0 & 1 \end{array}],M_{rt2}(\theta_{r})=[\begin{array}{cccc} 1 & 0 & 0 & \rho_{r}\sin\theta_{r}\\ 0 & 1 & 0 & 0\\ 0 & 0 & 1 & \rho_{r}-\rho_{r}\cos\theta_{r}\\ 0 & 0 & 0 & 1 \end{array}]$$ (38)

Then the equations of the tooth surfaces of helical rack and curvilinear rack can be expressed in *S*_*r*_ as

$$\left\{ \begin{array}{l} r_{r1}(u_{r},v_{r})=M_{rt}(v_{r})r_{t}(u_{r})={\left[\begin{array}{cccc} u_{r}-v_{r}\tan\beta_{r} & f(u_{r}) & v_{r} & 1 \end{array}\right]}^{T}\\ r_{r2}(u_{r},\theta_{r})=M_{rn}(\theta_{r})r_{t}(u_{r})={\left[\begin{array}{cccc} u_{r}+\rho_{r}\sin\theta_{r} & f(u_{r}) & \rho_{r}(1-\cos\theta_{r}) & 1 \end{array}\right]}^{T} \end{array}\right.$$ (39)

When the helix angle *β*_*r*_ is 0, it is a straight rack. The herringbone tooth can be obtained by the helical tooth. Moreover, *θ*_*r*_ can be represented by *v*_*r*_ as *θ*_*r*_ = arccos(1−*v*_*r*_/*ρ*_*r*_). Then the equations of the tooth surfaces of the four racks can be expressed by ***r***_*r*_(*u*_*r*_, *v*_*r*_) uniformly.

**Fig 17:**
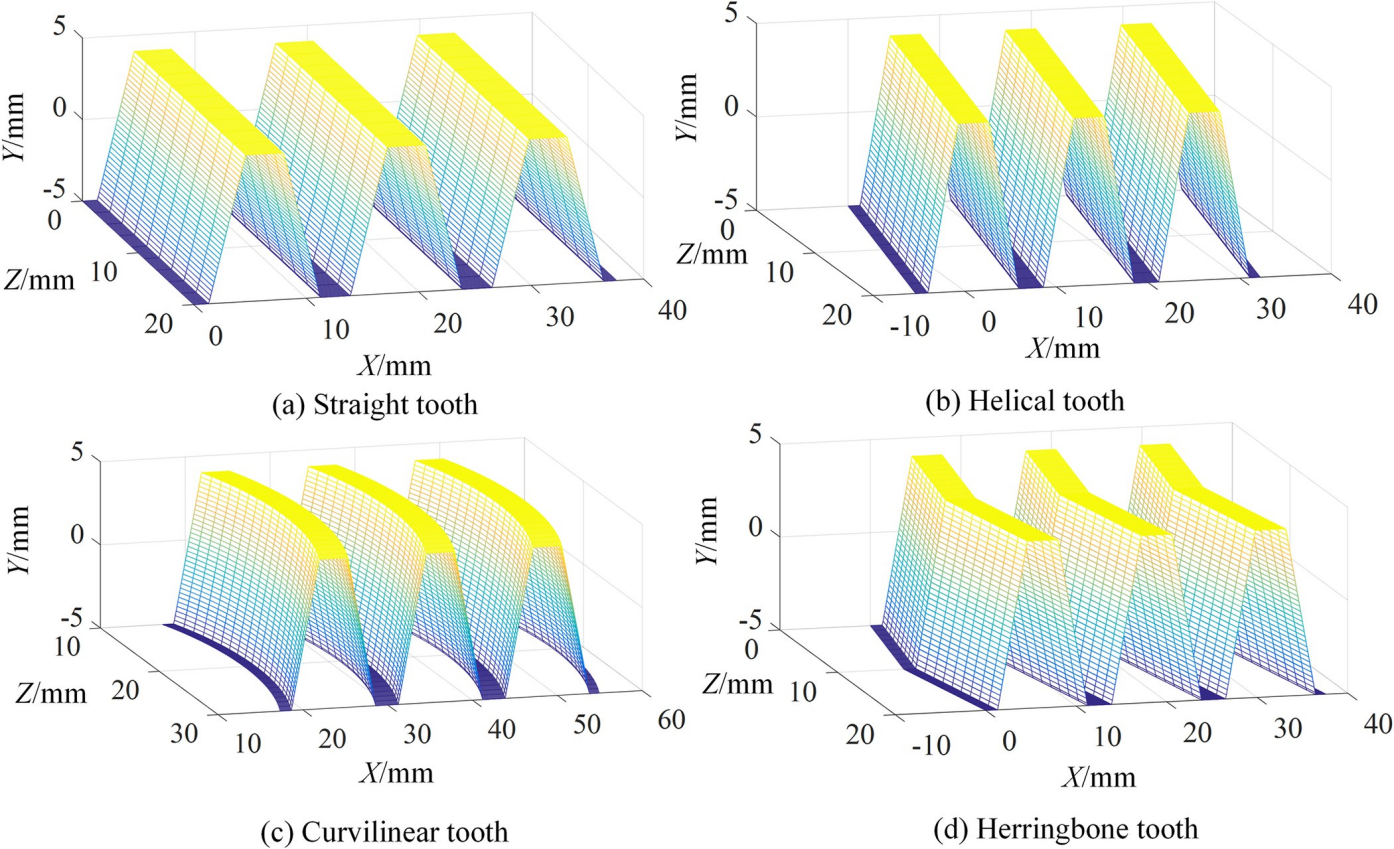
Tooth surfaces of rack generator with different tooth profiles.

Taking the structure parameters in Table 4, the tooth surfaces of rack generator with different tooth profiles were obtained, as shown in Fig 17.

**Table 4:** Structure parameters of rack generator.

Parameter	Module *m*_*r*_	Coefficient of addendum	Coefficient of bottom clearance	Pressure angle *α*_*r*1_, *α*_*r*2_	Tooth width	Helical angle *β*_*r*_	Arc radius *ρ*_*r*_
Value	4mm	1	0.25	20°	20mm	15°	20mm

#### Cylindrical generator

**Fig 18:**
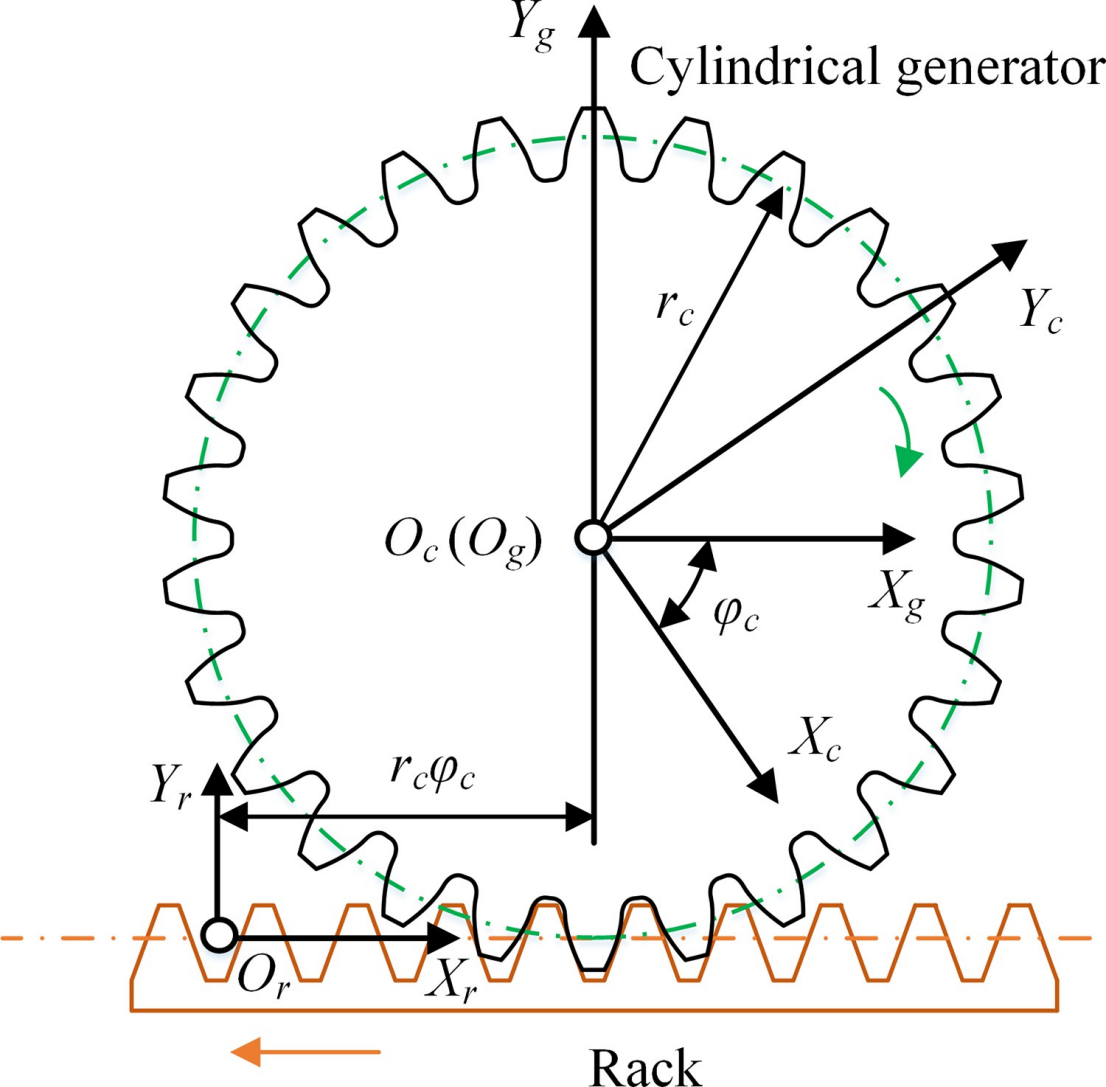
Generating process from rack to cylindrical generator.

The four kinds of cylindrical generator have the same generation process. Fig 18 is the generating process from rack to cylindrical generator. The coordinate system *S*_*r*_ is fixed with the rack. *S*_*g*_ (*O*_*g*_*−X*_*g*_*Y*_*g*_*Z*_*g*_), abbreviated as *S*_*g*_, is the fixed coordinate system of cylindrical generator. *S*_*c*_ (*O*_*c*_*−X*_*c*_*Y*_*c*_*Z*_*c*_), abbreviated as *S*_*c*_, is the follow-up coordinate system of cylindrical generator. *z*_*c*_ and *r*_*c*_ are the number of teeth and the radius of cylindrical generator respectively. *r*_*c*_ = *m*_*r*_*z*_*c*_/2. When the rotation angle of cylindrical generator is *φ*_*c*_ = *u*_*c*_/*r*_*c*_, the displacement of rack is *r*_*c*_*φ*_*c*_.

Based on the principle of spatial coordinate transformation, the transformation matrix from *S*_*r*_ to *S*_*c*_ is

$$M_{cr}=M_{cg}M_{gr}=[\begin{array}{cccc} \cos\varphi_{c} & -sin\varphi_{c} & 0 & -r_{c}(\varphi_{c}\cos\varphi_{c}-sin\varphi_{c})\\ sin\varphi_{c} & \cos\varphi_{c} & 0 & -r_{c}(\varphi_{c}\sin\varphi_{c}+\cos\varphi_{c})\\ 0 & 0 & 1 & 0\\ 0 & 0 & 0 & 1 \end{array}]$$ (40)

Then the equation of the tooth surface of cylindrical generator in *S*_*c*_ can be expressed as.


$$r_{c}(u_{r},v_{r})=M_{cr}r_{r}(u_{r},v_{r})$$ (41)

**Fig 19:**
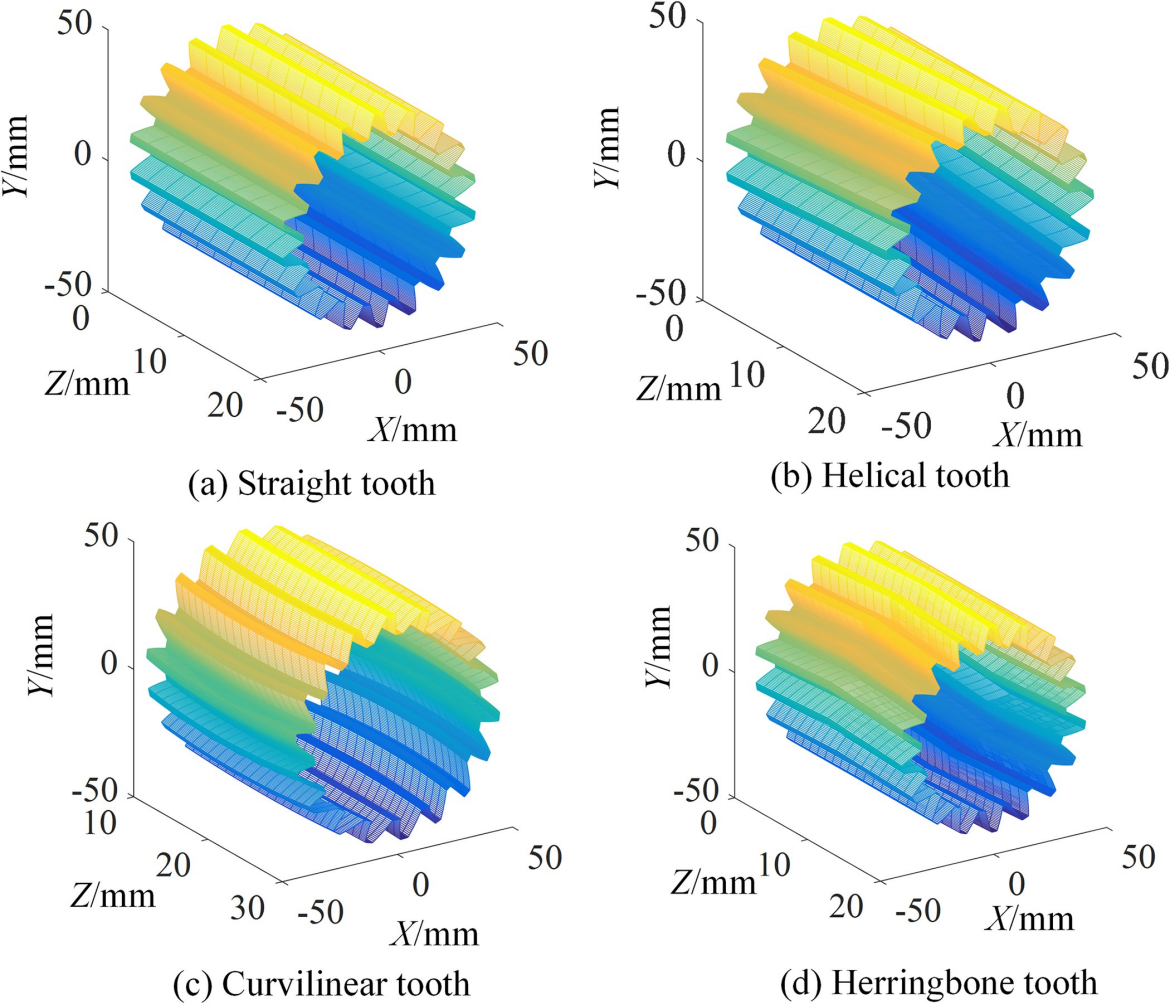
Tooth surfaces of cylindrical generator with different tooth profiles.

Taking the parameters in Table 4, the tooth surfaces of cylindrical generator with different tooth profiles were obtained, as shown in Fig 19.

#### Conical generator

**Fig 20:**
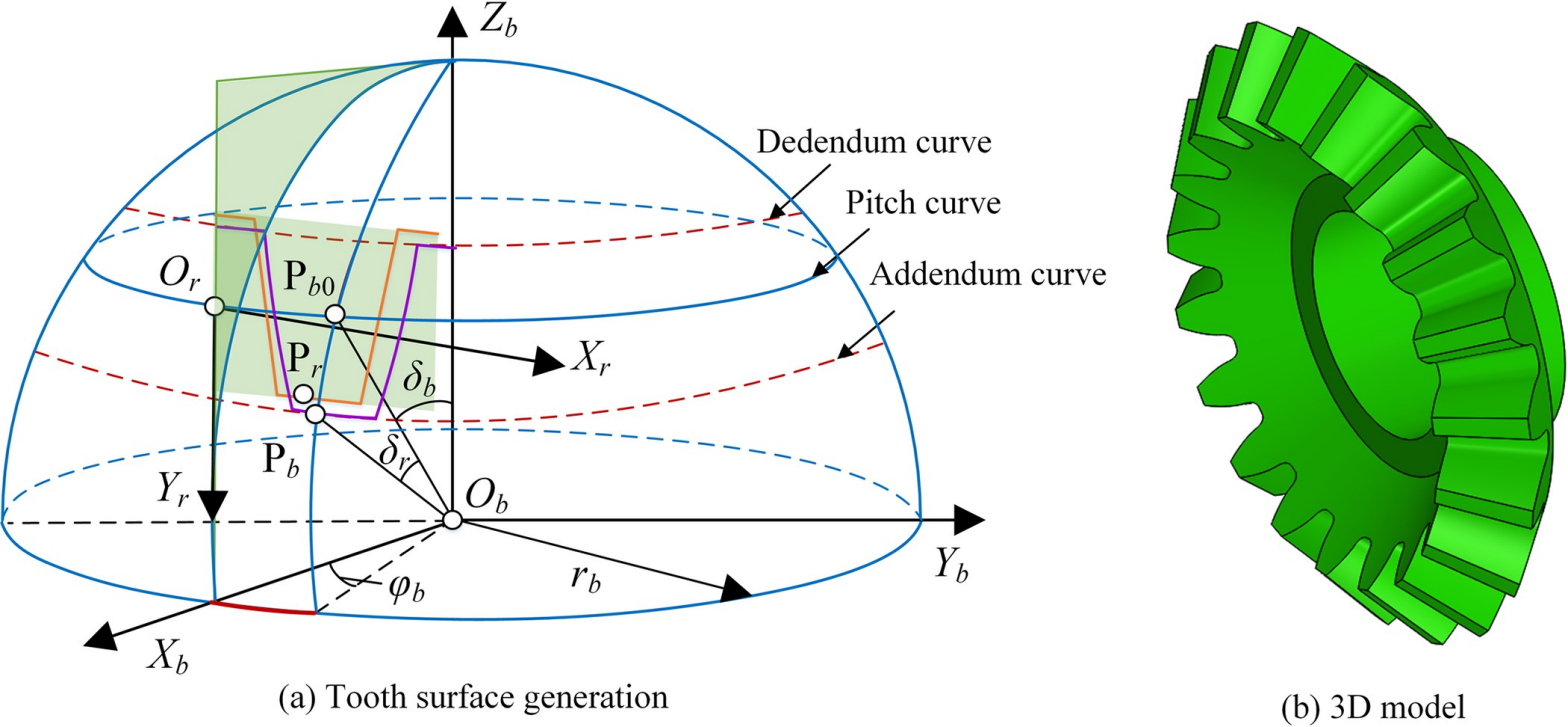
Tooth surface generation and 3D model of conical generator.

Fig 20(A) shows the conversion process from rack to conical generator. *S*_*b*_ (*O*_*b*_*−X*_*b*_*Y*_*b*_*Z*_*b*_), abbreviated as *S*_*b*_, is the coordinate system of conical generator. P_*r*_ is the point on the tooth profile of rack, and P_*b*_ is the corresponding point on the tooth profile of conical generator. P_*b*0_ is the intersection between the pitch curve of conical generator and the big circle passing through *O*_*r*_P_*b*0_. *δ*_*b*_ is the pitch angle of conical generator. *δ*_*r*_ and *φ*_*b*_ are the angles corresponding to arc P_*b*_P_*b*0_ and *O*_*r*_P_*b*0_ respectively. *r*_*b*_ is the spherical radius. The equation of the tooth surface of conical generator can be expressed as

$$r_{b}(u_{r},v_{r})={\left[v_{r}\sin(\delta_{b}+\delta_{r})\cos\varphi_{b},v_{r}\sin(\delta_{b}+\delta_{r})\sin\varphi_{b},v_{r}\cos(\delta_{b}+\delta_{r})\right]}^{T}$$ (42)

Where, $\delta_{r}=f(u_{r})/r_{b},\varphi_{b}=u_{r}/r_{b}$. *v*_*r*_ is the tooth width parameter and its range is [*r*_*b*_–*B*_*b*_, *r*_*b*_]. *B*_*b*_ is the tooth width.

Taking the parameters of the straight rack in Table 4, the 3D model of conical generator can be obtained by Eq (42), as shown in Fig 20(B).

### Motion process of generator

#### Motion coordinate system of generator

**Fig 21:**
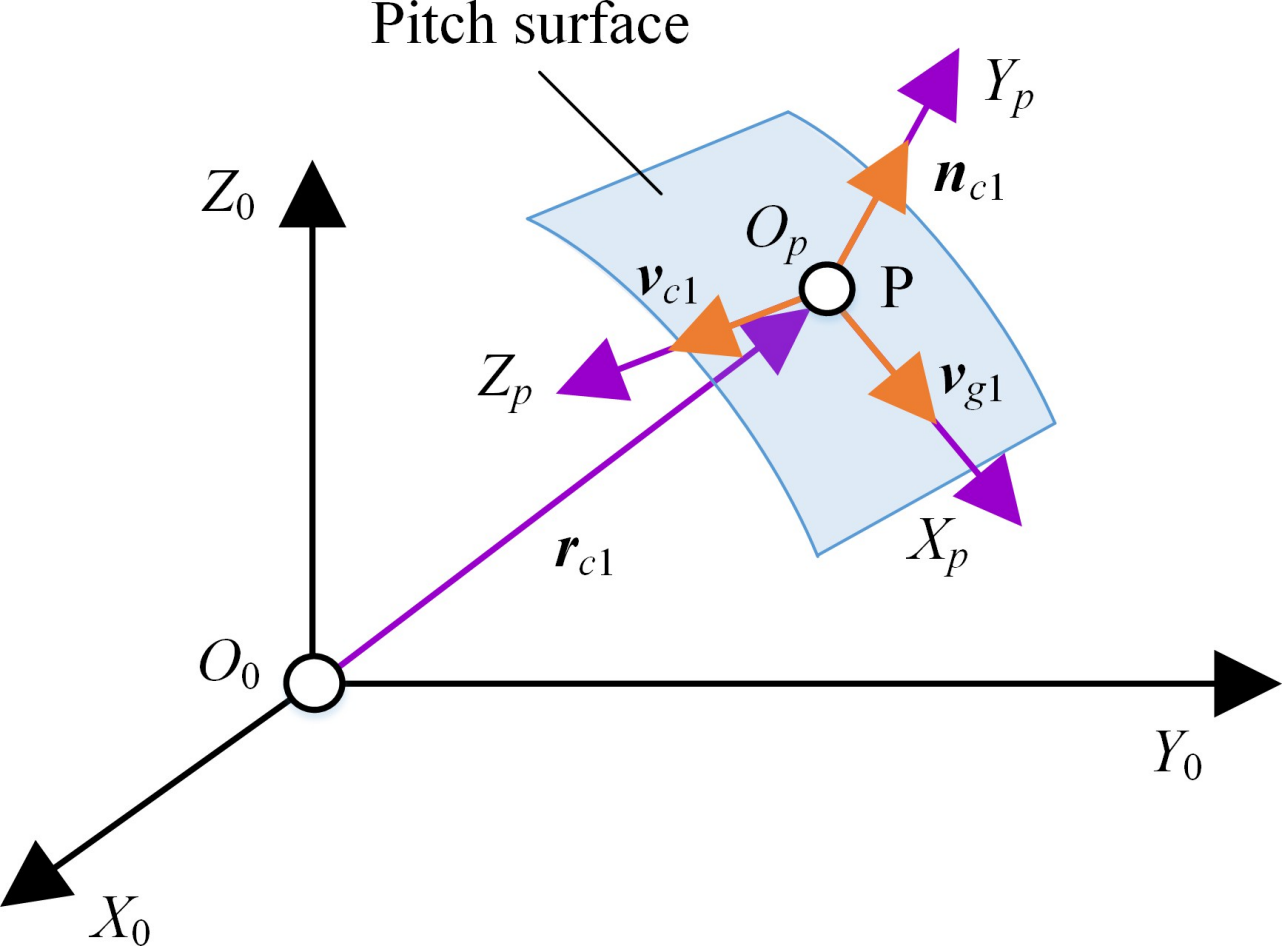
Motion coordinate system of generator.

Based on the meshing theory, the generating principle can be understood as ensuring that one tangent vector of the tooth surface of generator at the meshing point is in the same direction with the relative speed. Thus, the motion coordinate system and position conversion relationship of generator can be used to obtained the motion relationship between generator and SNCG. As shown in Fig 21, at the meshing point P, the reference coordinate system *S*_*p*_ (*O*_*p*_*−X*_*p*_*Y*_*p*_*Z*_*p*_) of generator can be established on the common tangent plane of the pitch surfaces of SNCG.

For convenience, in *S*_0_, the position vector ***r***_*pl*_ of point Q was represented as ***r***_*c*1_. The velocity ***v***_*p*1*l*_ of gear 1 relative to the instant screw axis was represented as ***v***_*c*1_, and the common normal vector ***n***_*pl*_ of the two gears is represented as ***n***_*c*1_.


$$\left\{r_{c1},n_{c1},v_{c1}\}=\{r_{pl},n_{pl},v_{p1l}\right\}$$ (43)

Axis *O*_*p*_*Z*_*p*_ is along the direction of ***v***_*c*1_, axis *O*_*p*_*Y*_*p*_ is along the direction of ***n***_*c*1_, and the direction of axis *O*_*p*_*X*_*p*_, that is, the direction of the velocity of generator, can be determined by the right-hand rule. Then the velocity of generator can be expressed as

$$v_{g1}=|v_{g1}|{\bar{v}}_{g1}=v_{p1}\cdot {\bar{v}}_{c1}\cdot ({\bar{n}}_{c1}\times {\bar{v}}_{c1})$$ (44)

Where, ${\bar{v}}_{g1},{\bar{n}}_{c1}$ and ${\bar{v}}_{c1}$ are the unit vectors of ***v***_*g*1_, ***n***_*c*1_ and ***v***_*c*1_ respectively.

The transformation matrix from *S*_*p*_ to *S*_0_ is

$$M_{0p}=[\begin{array}{cccc} i_{0}\cdot i_{p} & i_{0}\cdot j_{p} & i_{0}\cdot k_{p} & x_{p}\\ j_{0}\cdot i_{p} & j_{0}\cdot j_{p} & j_{0}\cdot k_{p} & y_{p}\\ k_{0}\cdot i_{p} & k_{0}\cdot j_{p} & k_{0}\cdot k_{p} & z_{p}\\ 0 & 0 & 0 & 1 \end{array}]=[\begin{array}{cccc} {\bar{v}}_{g1} & {\bar{n}}_{c1} & {\bar{v}}_{c1} & r_{c1}\\ 0 & 0 & 0 & 1 \end{array}]$$ (45)

Where, $i_{0}={\left[1,0,0\right]}^{T},j_{0}={\left[0,1,0\right]}^{T},k_{0}={\left[0,0,1\right]}^{T},i_{p}={\bar{v}}_{g1},j_{p}={\bar{n}}_{c1},k_{p}={\bar{v}}_{c1}$ are the unit vectors of the coordinate axes of *S*_0_ and *S*_*p*_, respectively (*x*_*p*_,*y*_*p*_,*z*_*p*_) is the coordinate of point P in *S*_0_.

Similarly, in order to facilitate the derivation of the generation relationship of gear 2, the vectors *v*_*g*2_, *n*_*c*2_, *v*_*c*2_ and *r*_*c*2_ can be defined in *S*_*f*_ and obtained from Eq (46).


$$\left\{v_{g2},n_{c2},v_{c2},r_{c2}\}=\{L_{f0}v_{g1},L_{f0}n_{c1},L_{f0}v_{c1},M_{f0}r_{c1}\right\}$$ (46)

The transformation matrix from *S*_*p*_ to *S*_*f*_ is

$$M_{fp}=[\begin{array}{cccc} {\bar{v}}_{g2} & {\bar{n}}_{c2} & {\bar{v}}_{c2} & r_{c2}\\ 0 & 0 & 0 & 1 \end{array}]$$ (47)

Where, ${\bar{v}}_{g2},{\bar{n}}_{c2}$ and ${\bar{v}}_{c2}$ are the unit vectors of ***v***_*g*2_, ***n***_*c*2_ and ***v***_*c*2_ respectively. ***r***_*c*2_ is the position vector of Q in *S*_*f*_.

#### Position conversion of generator

**Fig 22:**
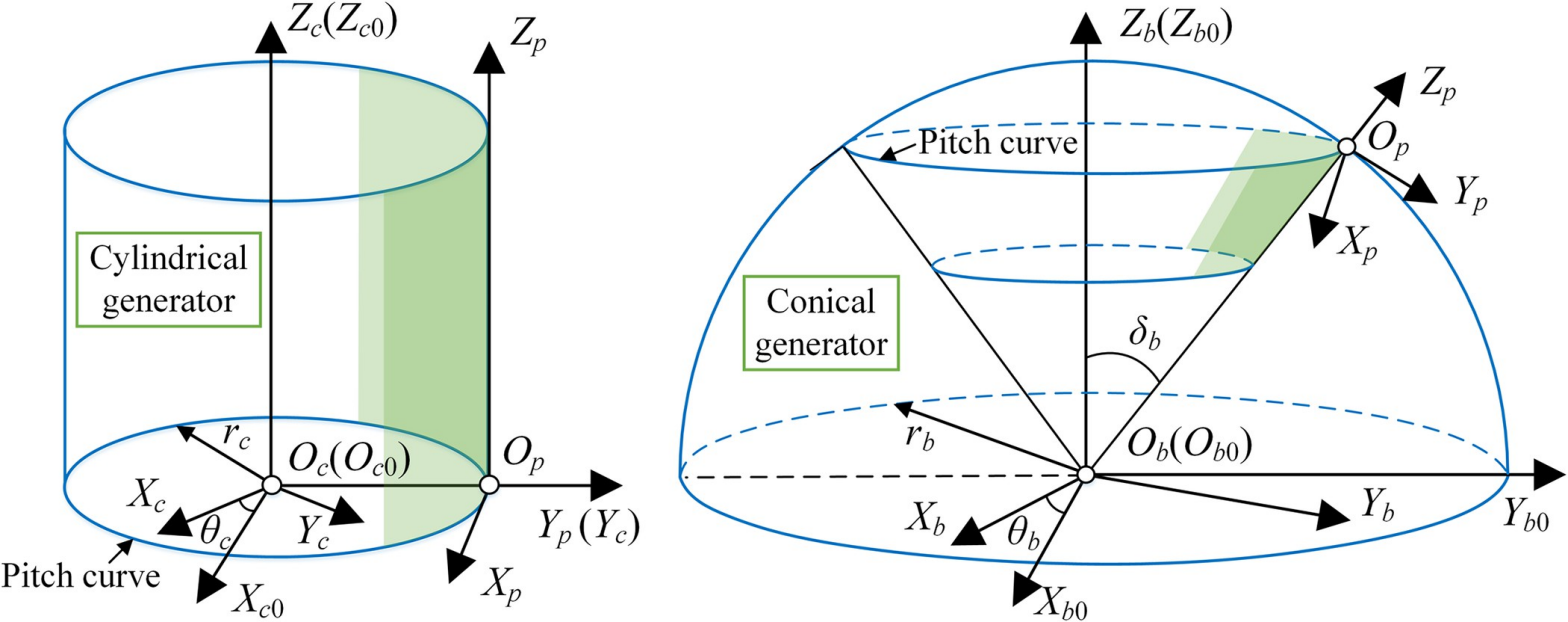
Position conversion of generator.

Fig 22 shows the position conversion from the coordinate system of the tooth surface to the reference coordinate system *S*_*p*_. The corresponding parameters have the same meaning as before. *S*_*c*0_ (*O*_*c*0_ *–X*_*c*0_*Y*_*c*0_*Z*_*c*0_) and *S*_*b*0_ (*O*_*b*0_ *–X*_*b*0_*Y*_*b*0_*Z*_*b*0_), abbreviated as *S*_*c*0_ and *S*_*b*0_, are the auxiliary coordinate systems of cylindrical generator and conical generator, respectively.

The transformation matrices from *S*_*c*_ and *S*_*b*_ to *S*_*p*_ are

$$M_{pc}=[\begin{array}{cccc} \cos\theta_{c} & \sin\theta_{c} & 0 & 0\\ -\sin\theta_{c} & \cos\theta_{c} & 0 & -r_{c}\\ 0 & 0 & 1 & 0\\ 0 & 0 & 0 & 1 \end{array}],M_{pb}=[\begin{array}{cccc} \cos\theta_{b} & \sin\theta_{b} & 0 & 0\\ -\cos\delta_{b}\sin\theta_{b} & \cos\delta_{b}\cos\theta_{b} & -\sin\delta_{b} & 0\\ -\sin\delta_{b}\sin\theta_{b} & \sin\delta_{b}\cos\theta_{b} & \cos\delta_{b} & -r_{b}\\ 0 & 0 & 0 & 1 \end{array}]$$ (48)

Where, *θ*_*c*_ and *θ*_*c*_ are the rotation angles of cylindrical generator and conical generator during the generation motion respectively. $\theta_{c}=\int_{0}^{\theta_{1}}|v_{g1}|/r_{c}d\theta ,\theta_{b}=\int_{0}^{\theta_{1}}|v_{g1}|/(r_{b}\sin\delta_{b})d\theta$. *δ*_*b*_ is the pitch angle of conical generator.

### Tooth surface of SNCG

For convenience, *S*_*c*_ and *S*_*b*_ are collectively referred to as *S*_*G*_. ***r***_*c*_(*u*_*r*_,*v*_*r*_) and ***r***_*b*_(*u*_*r*_,*v*_*r*_) are collectively referred to as ***r***_*G*_(*u*_*r*_,*v*_*r*_). ***M***_*pc*_ and ***M***_*pb*_ are collectively referred to as ***M***_*pG*_. Based on the motion process of generator, the transformation matrices from *S*_*G*_ to *S*_1_ and *S*_2_ can be obtained as

$$\left\{ \begin{array}{l} M_{1G}(\theta )=M_{10}(\theta )M_{0G}(\theta )=M_{10}(\theta )M_{0p}(\theta )M_{pG}(\theta )\\ M_{2G}(\theta )=M_{2f}(\theta )M_{fG}(\theta )=M_{2f}(\theta )M_{fp}(\theta )M_{pG}(\theta ) \end{array}\right.$$ (49)

Where, *θ* is *θ*_1_ or *θ*_2_. ***M***_0*G*_(*θ*) and ***M***_*fG*_(*θ*) are the transformation matrices from *S*_*G*_ to *S*_0_ and *S*_*f*_, respectively.

Then the tooth surfaces of the two gears can be obtained by Eq (50).


$$\left\{ \begin{array}{l} r_{1}(\theta ,u_{r},v_{r})=M_{1G}(\theta )r_{G}(u_{r},v_{r})\\ r_{2}(\theta ,u_{r},v_{r})=M_{2G}(\theta )r_{G}(u_{r},v_{r})\\ f_{1G}(\theta ,u_{r},v_{r})=f_{2G}(\theta ,u_{r},v_{r})=f_{12}(\theta ,u_{r},v_{r})=0 \end{array}\right.$$ (50)

Where, *f*_1*G*_(*θ*,*u*_*r*_,*v*_*r*_), *f*_2*G*_(*θ*,*u*_*r*_,*v*_*r*_) and *f*_12_(*θ*,*u*_*r*_,*v*_*r*_) are the equations of meshing between generator and gear 1, generator and gear 2, and gear 1 and gear 2 respectively.


$$\left\{ \begin{array}{l} f_{1G}(\theta ,u_{r},v_{r})=L_{0G}(\theta )n_{G}(u_{r},v_{r})\cdot (v_{g1}-v_{p1})\\ f_{2G}(\theta ,u_{r},v_{r})=L_{0G}(\theta )n_{G}(u_{r},v_{r})\cdot (v_{g2}-v_{p2})\\ f_{12}(\theta ,u_{r},v_{r})=L_{0G}(\theta )n_{G}(u_{r},v_{r})\cdot v_{p12} \end{array}\right.$$ (51)

Where, ***L***_0*G*_(*θ*) is a submatrix consisting of the first three rows and three columns of ***M***_0*G*_(*θ*). ***n***_*G*_(*u*_*r*_,*v*_*r*_) is the normal vector of the tooth surface of generator in *S*_*G*_, which can be obtained by Eq (52).


$$n_{G}(u_{r},v_{r})=\frac{\partial r_{G}(u_{r},v_{r})}{\partial u_{r}}\times \frac{\partial r_{G}(u_{r},v_{r})}{\partial v_{r}}$$ (52)

**Fig 23:**
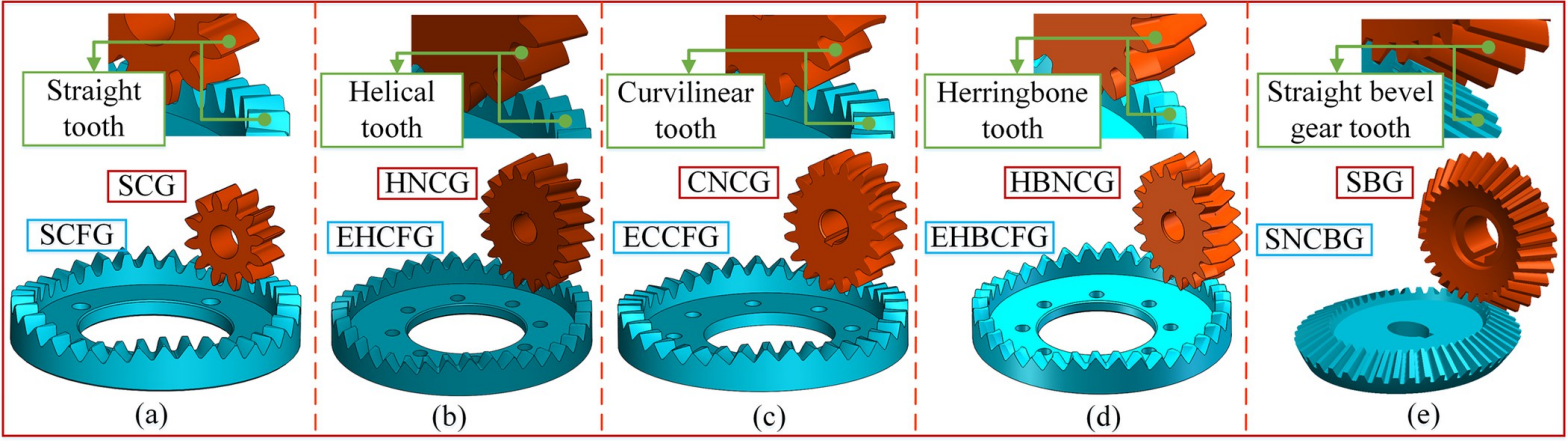
Tooth surface and 3D model of SNCG.

According to the above method, taking the parameters in Table 5, the tooth surface and 3D model of SNCG can be obtained, as shown in Fig 23.

**Table 5:** Structural parameters of SNCG.

Type	Gear pair	Number of teeth *Z*_1_/*Z*_2_	Module	Coefficient of addendum	Coefficient of bottom clearance	Order *n*_1_	Order *n*_2_	Eccentric distance
I	SCFGP	18/36	4mm	1.0	0.25	/	4	/
II	EHCFGP	36/18				4	2	5mm
	ECCFGP							
	EHBCFGP							
	SNCBGP	44/30				2	/	/

As can be seen from Fig 23, the tooth surfaces of SNCG with different tooth profiles and structural forms are consistent with the expectation, which shows the rationality and correctness of screw analysis method for SNCG compound transmission.

## Experiments

In order to further verify the correctness of the theoretical analysis, the finished products of SNCGP were obtained by machining, and experiments, including tooth surface measurement, transmission ratio measurement and axial displacement measurement, were carried out. Specifically, tooth surface measurement is used to verify the correctness of theoretical model of tooth surface. Transmission ratio measurement and axial displacement measurement are used to verify the correctness of compound transmission characteristics. All experiments are combined to verify the rationality and correctness of the screw analysis method. In addition, for type II, the compound motion law of SNCBGP is similar to that of ECFGP, in order to avoid repetition, only ECFGP was taken as the experimental object in the next experiments.

### SNCG machining

**Fig 24:**
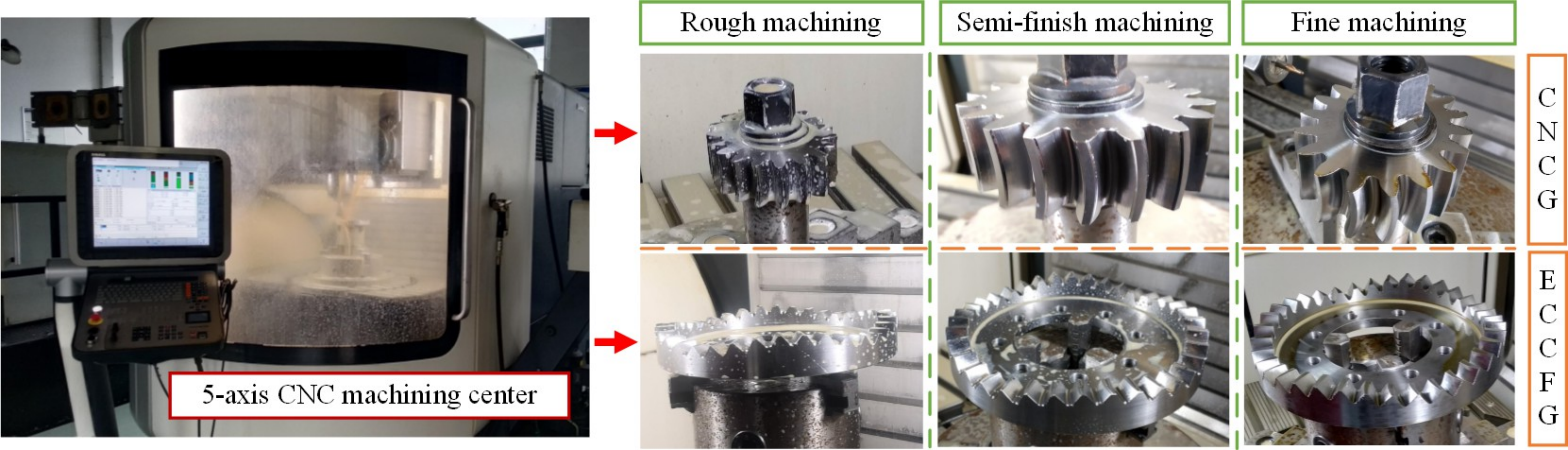
Machining process of ECCFGP.

Based on the 3D solid model, taking the material 20CrMnTi, SNCG can be produced by five-axis CNC milling, and the model number of the equipment is DMU60monoBLOCK (produced by GILDEMEISTER-Group). Taking ECCFGP as an example, the machining process was shown in Fig 24.

**Fig 25:**
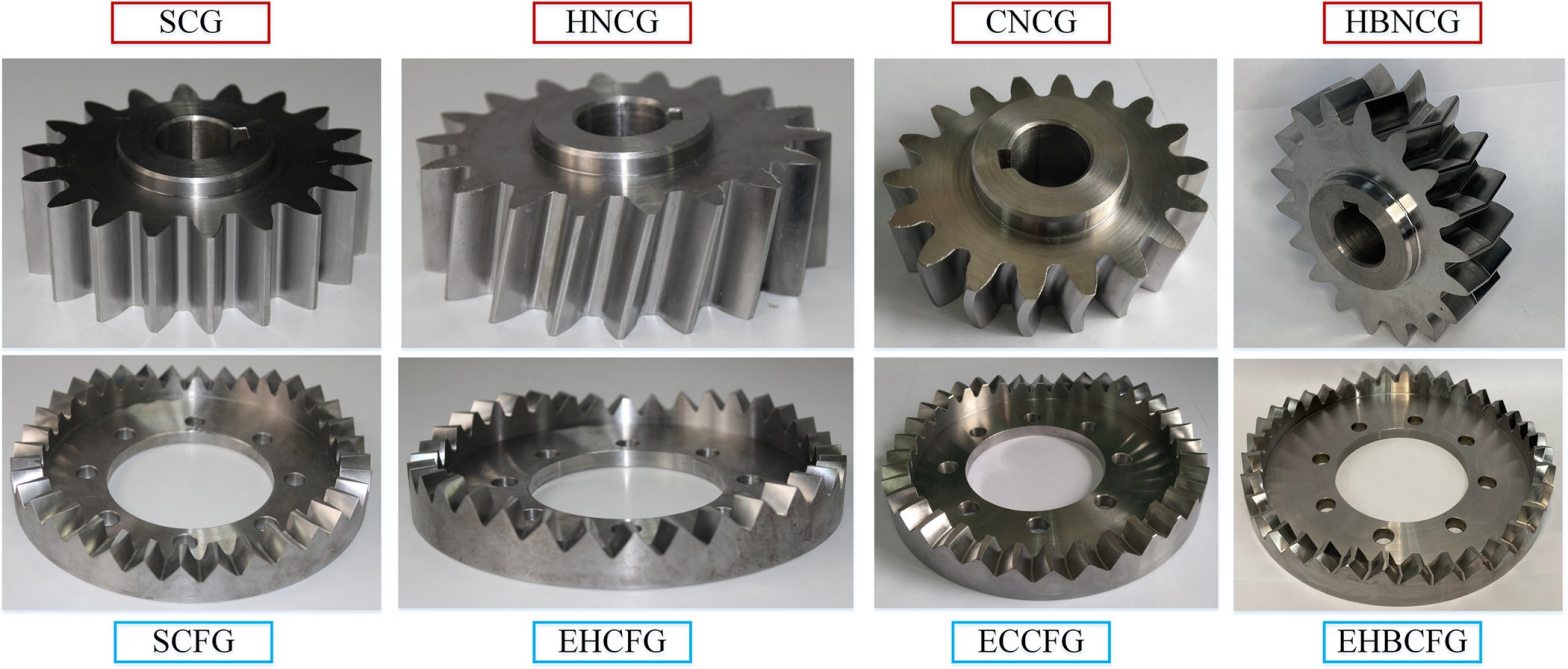
Finished products of SNCG.

The machining process mainly includes three parts: rough machining, semi-finish machining and fine machining. Set different processing parameters according to the shape of the gear teeth. Before machining, tools and blanks need to be installed and calibrated. The accurate tooth surfaces of CNCG and ECCFG were obtained by material removal without a secondary clamping. The finished products of SNCG were shown in Fig 25, which will be used for subsequent experiments.

### Tooth surface measurement

**Fig 26:**
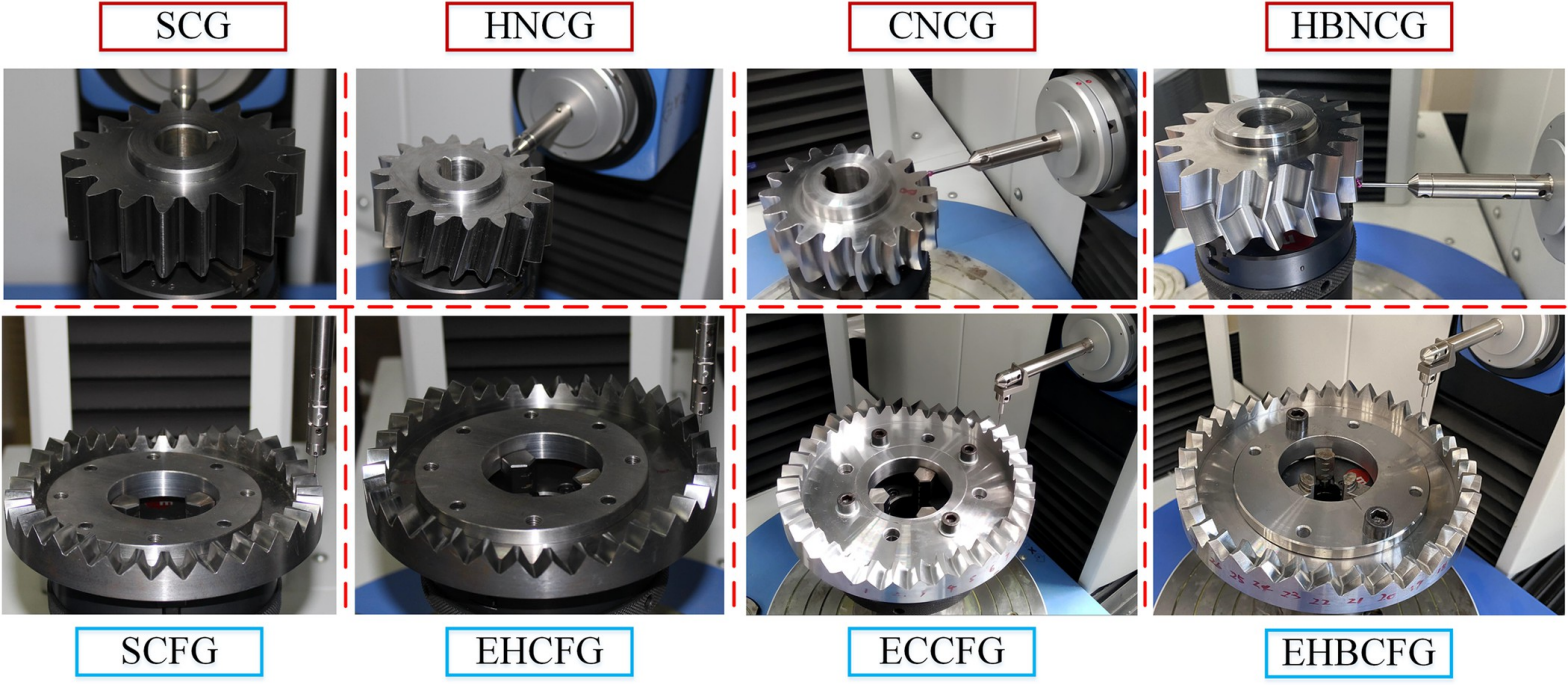
Tooth surface measurement of each gear.

To verify the correctness of the theoretical model, tooth surface measurement was carried out, shown in Fig 26.

The P26 Automatic CNC Control Gear Testing Center (produced by Klingenberg Company in Germany) was used to carry out the tooth surface measurement in layers. The interval of each layer is 1mm, and the radius of the probe is 0.2mm.

**Fig 27:**
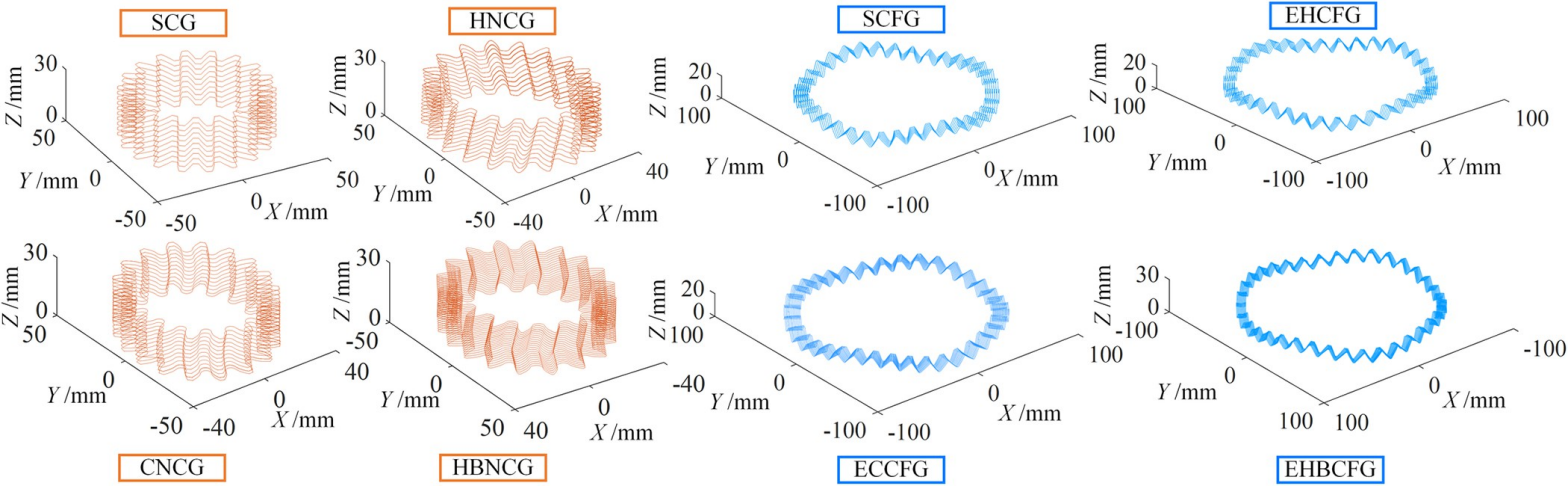
Measurement curve of the tooth surface of each gear.

After post-processing the measured data, the measurement curve of each gear can be obtained, as shown in Fig 27.

**Fig 28:**
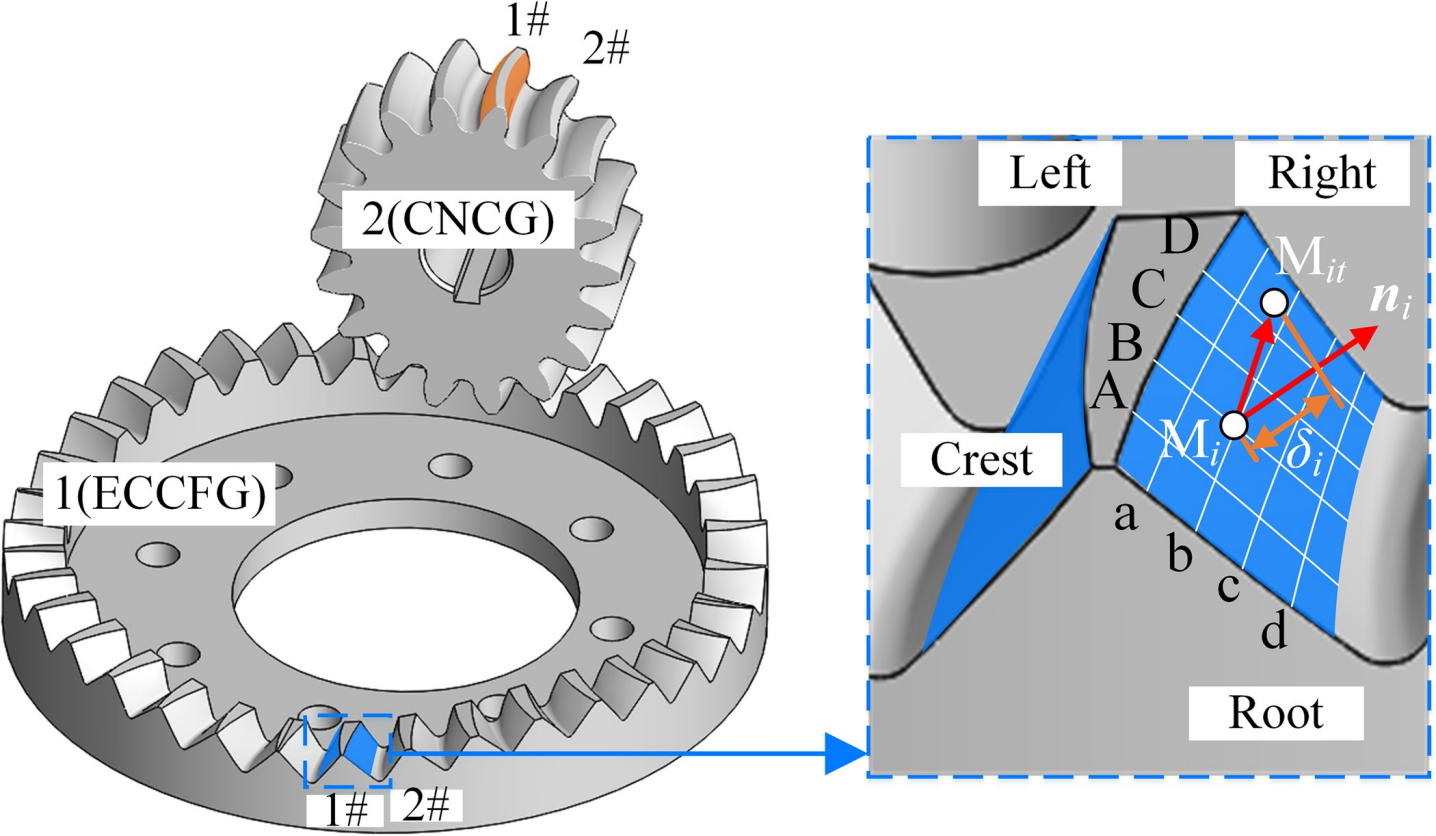
Calculation method of tooth surface error.

As shown in Fig 28, the measured curve was divided into blocks. Taking four layers as an example, the horizontal direction is a, b, c and d respectively. The vertical direction is A, B, C and D respectively. ***r***_*i*_ is the position vector of the theoretical point M_*i*_, ***r***_*it*_ is the position vector of the measuring point M_*it*_. ***n***_*i*_ is the normal vector of the tooth surface at point M_*i*_. The normal error *δ*_*i*_ of the tooth surface at this point is

$$\delta_{i}=(r_{it}-r_{i})\cdot n_{i}$$ (53)

When *δ*_*i*_>0, M_*it*_ is outside the theoretical tooth surface, indicating an insufficient cutting. When *δ*_*i*_<0, M_*it*_ is inside the theoretical tooth surface, indicating an excessive cutting.

**Fig 29:**
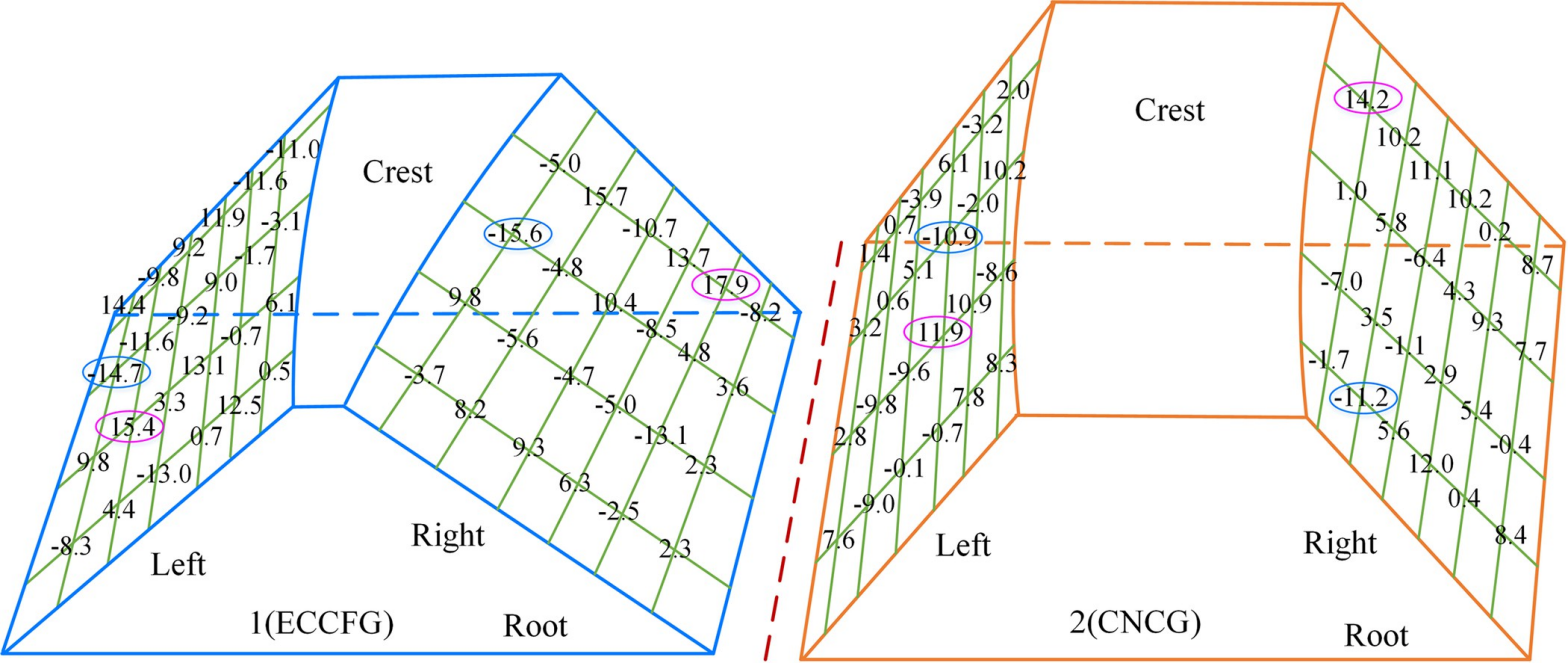
Tooth surface error of tooth 1#.

Taking tooth 1# of ECCFGP as an example, calculate the tooth surface errors of each gear respectively, and the results were shown in Fig 29.

The error of each tooth surface is close. Specifically, for tooth 1# of ECCFG, the variation ranges of the errors of the left and right tooth surface are –14.7μm~15.4μm and –15.6μm~17.9μm, respectively. For tooth 1# of CNCG, the variation ranges of the errors of the left and right tooth surface are –10.9μm~11.9μm and –11.2μm~14.2μm, respectively. The maximum of the errors of CNCG is smaller than that of ECCFG, and the maximum errors of the right tooth surfaces of the two gears are slightly higher than that of the left tooth surfaces.

**Fig 30:**
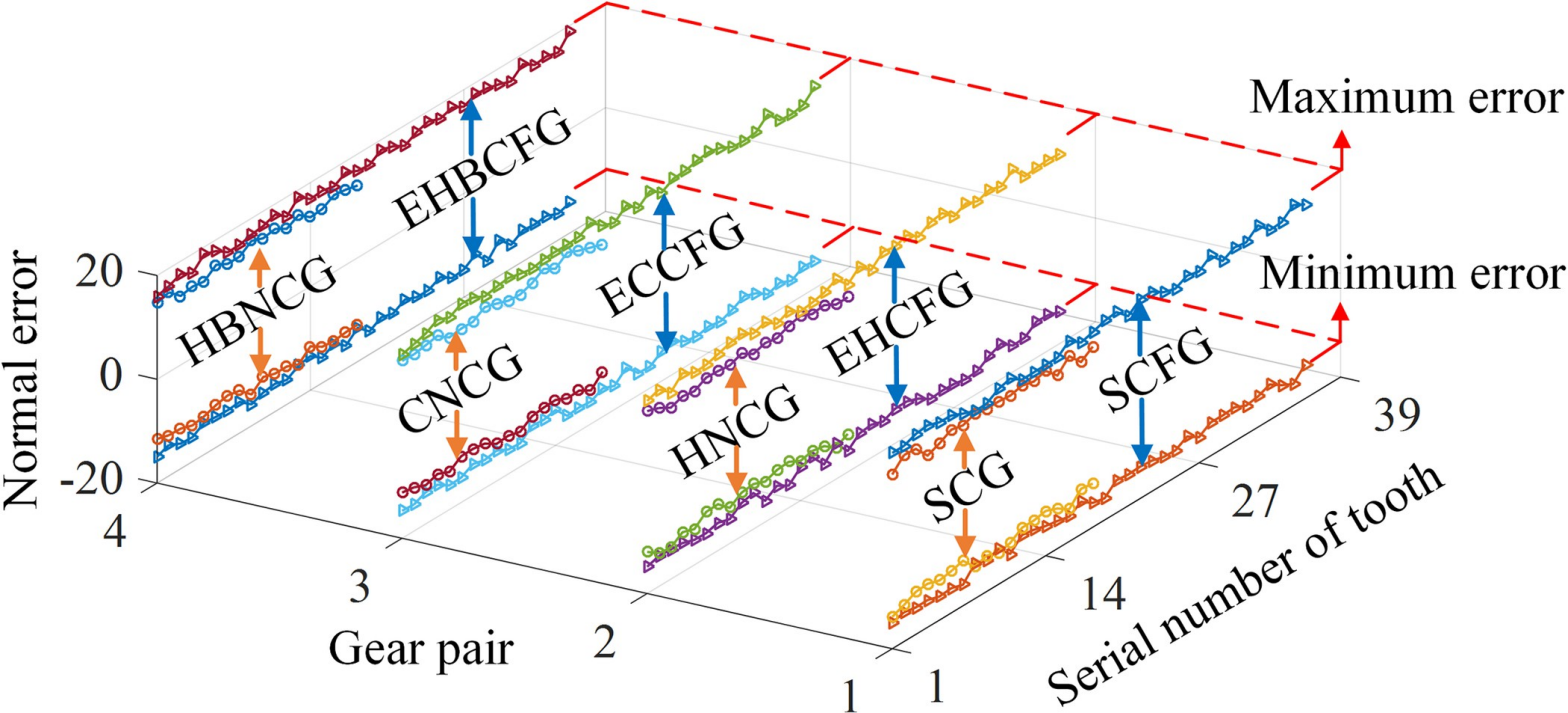
Maximum and minimum of tooth surface error of each tooth.

Then the tooth surface errors of all teeth of each gear were analyzed, and the maximum and minimum were obtained, as shown in Fig 30 and Table 6.

**Table 6:** Maximum and minimum of the tooth surface error.

Error	SCFGP		EHCFGP		ECCFGP		EHBCFGP	
	SCG	SCFG	HNCG	EHCFG	CNCG	ECCFG	HBNCG	EHBCFG
Maximum /μm	15.9	18.9	15.7	19.0	15.8	19.2	15.6	19.1
Minimum /μm	-13.8	-15.9	-13.9	-16.1	-13.6	-16.3	-13.8	-16.2

As can be seen, the absolute values of the maximum error and the minimum error of SCG and ECCFG are slightly larger than those of SCG and NCG respectively. This is because the tooth surfaces of SCG and ECCFG are more complex, and the curvature of tooth surface changes greatly, resulting in a larger tooth surface error. The tooth surface errors of each SNCG are within a reasonable variation range.

### Transmission experiment

**Fig 31:**
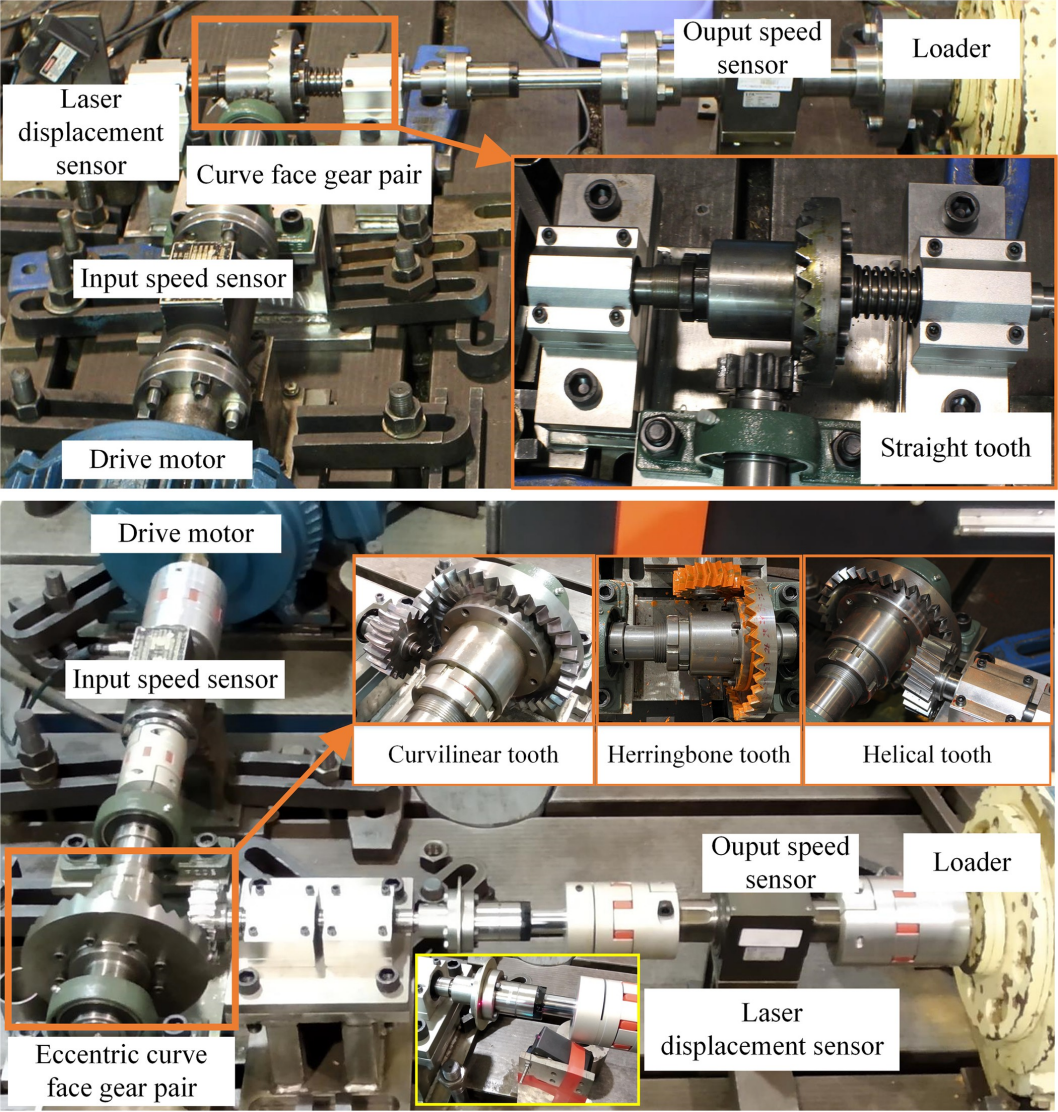
Transmission experiments of SNCG compound transmission. (a) Type I, (b) Type II.

In order to verify the correctness of the theoretical method and compound transmission characteristics, experimental test platforms were built respectively, as shown in Fig 31 (A) and 31(B). The input and output speeds were measured by the speed sensor, so that the transmission ratio can be calculated. The axial displacement of the driven gear was measured by a laser displacement sensor. The experimental conditions and parameters are shown in Table 7.

**Table 7:** Experimental conditions and parameters.

Parameter	Input speed	Load	Sampling frequency of speed sensor	Sampling frequency of laser displacement sensor
Value	200rpm	20Nm	4800kHz	200Hz

#### Transmission ratio comparison

**Fig 32:**
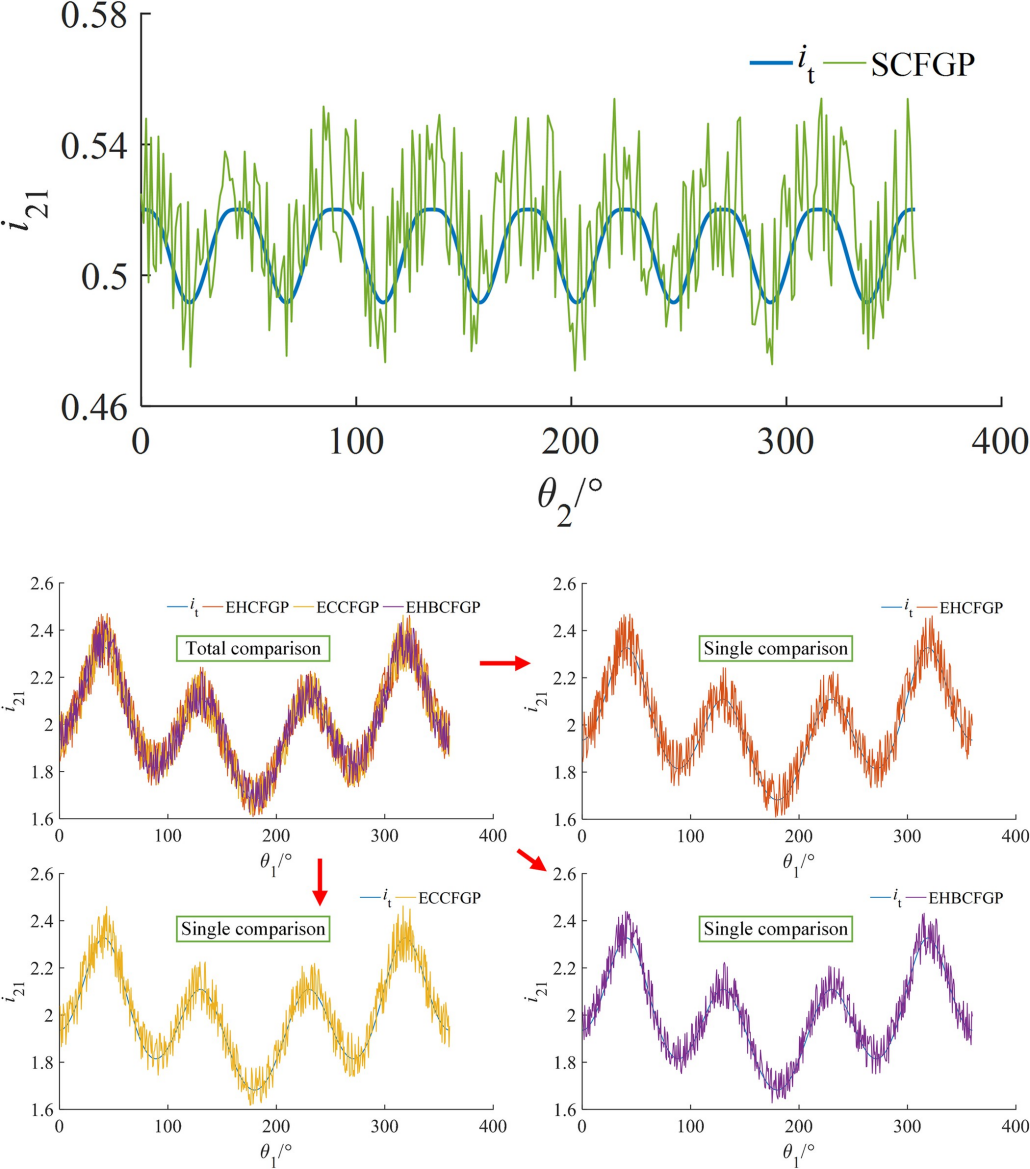
Comparison between theoretical and experimental transmission ratios. (a) Type I, (b) Type II.

The experimental values and the theoretical values of transmission ratios were compared and analyzed, as shown in Fig 32. The calculation method of the relative errors was shown in Eq (54), and results were shown in Table 8.


$$e_{i}=|i_{e}-i_{t}|/i_{t}\times 100\%$$ (54)

**Table 8:** Maximum error between theoretical and experimental values of transmission ratios.

Transmission ratio error	Type I	Type II		
	SCFGP	EHCFGP	ECCFGP	EHBCFGP
Maximum	6.8%	6.5%	5.7%	5.5%

Where, *i*_e_ and *i*_t_ represent the measured curve and theoretical curve of transmission ratio respectively.

As can be seen from Fig 32 and Table 8, the measured curves are consistent with the theoretical curves, and the corresponding maximum errors are 6.8%, 6.5%, 5.7% and 5.5% respectively. The maximum errors of SCFGP and EHCFGP are similar and larger than those of ECCFGP and EHBCFGP. The reason is that for SCFGP and EHCFGP, the axial reciprocating of the driven gear was realized by the spring force, which is easy to cause a speed error, while ECCFGP and EHBCFGP realize the axial reciprocating of the driven gear by their own tooth structure, and there is no such a speed error. In addition, for SCFGP, the number of change cycles of the transmission ratio is larger, resulting in a larger speed fluctuation and finally a larger error.

#### Axial displacement comparison

**Fig 33:**
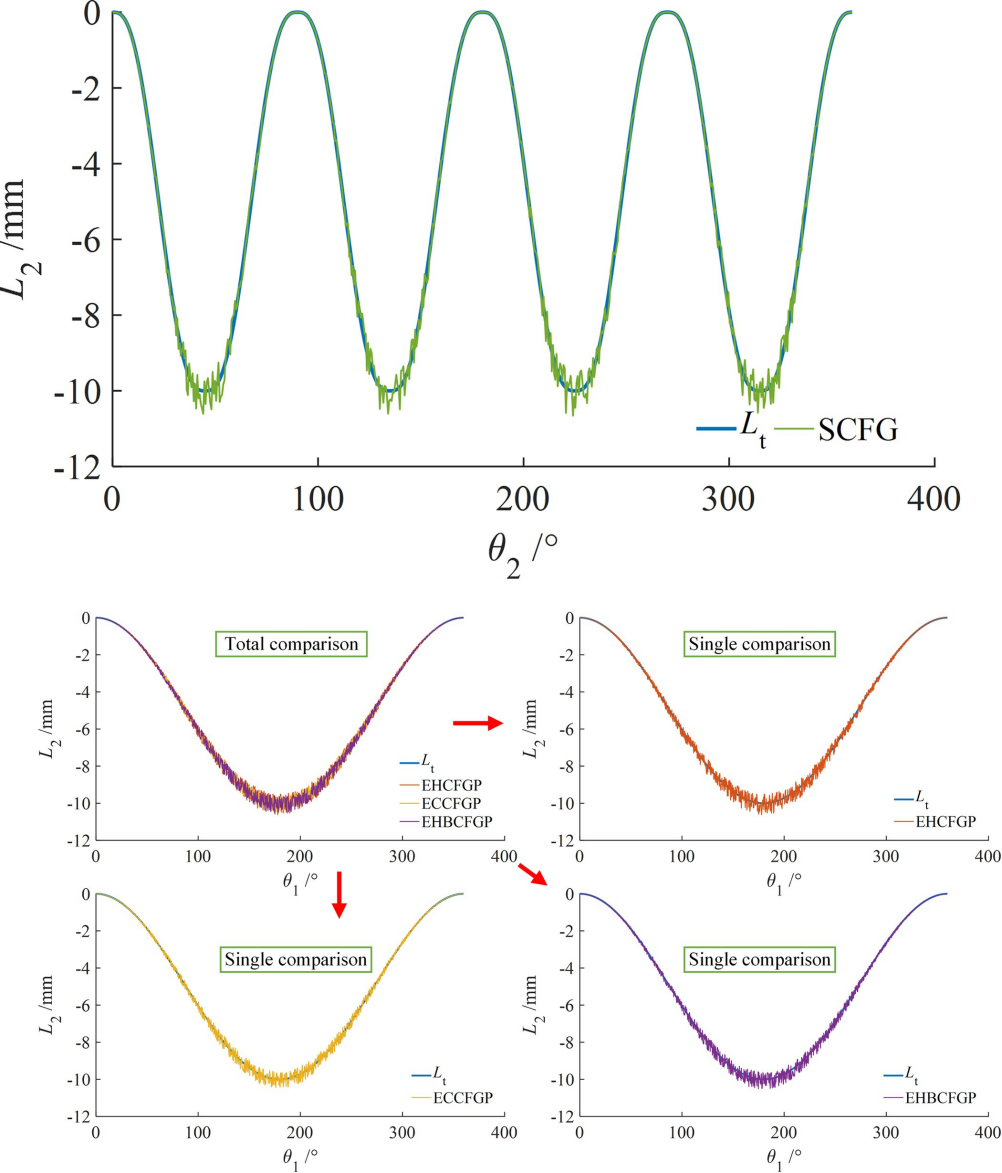
Comparison between theoretical and experimental axial displacements. (a) Type I, (b) Type II.

Similarly, the experimental values and the theoretical values of axial displacements were compared and analyzed, as shown in Fig 33. The calculation method of the relative errors was shown in Eq (55), and the results were shown in Table 9.


$$e_{L}=|L_{e}-L_{t}|/L_{t}\times 100\%$$ (55)

Where, *L*_e_ and *L*_t_ represent the measured curve and the theoretical curve of axial displacement respectively.

**Table 9:** Maximum error between theoretical and experimental values of axial displacements.

Axial displacement error	Type I	Type II		
	SCFGP	EHCFGP	ECCFGP	EHBCFGP
Maximum	6.7%	6.3%	5.1%	5.4%

As can be seen from Fig 33 and Table 9, the design amplitude of axial displacement is 10mm, the measured curves are consistent with the theoretical curves, and the maximum errors are 6.7%, 6.3%, 5.1% and 5.4% respectively. The maximum errors of SCFGP and EHCFGP are similar and larger than those of ECCFGP and EHBCFGP. Similarly, the reason is that for SCFGP and EHCFGP, the axial reciprocating of the driven gear was realized by the spring force, which is easy to cause an axial runout, while ECCFGP and EHBCFGP realize the axial reciprocating of the driven gear by the tooth structure, and there is no such an axial displacement error. Moreover, for SCFGP, the number of change cycles of the transmission ratio is larger, resulting in a larger axial displacement fluctuation and finally a larger error.

Considering the machining error, installation error and assembly error of the test platform, the errors of transmission ratio and axial displacement of each SNCGP are within a reasonable range. Through the tooth surface measurement and transmission experiment, the correctness of the screw analysis method for SNCG compound motion was effectively verified, which laid a theoretical foundation for the further study.

## Conclusions

Through the study on the screw analysis method and experiment of SNCG compound motion, the following conclusions can be obtained.

(1) SNCGP can realize the compound transmission with a variable transmission ratio between intersecting axes, including two categories named speed reduction and speed increase. Based on the meshing theory and screw theory, a screw analysis method was proposed and the screw geometry characteristics of compound motion were analyzed in detail. Compared with the traditional vector analysis method, this screw analysis method can more intuitively and uniformly reflect the principle of the compound motion, and can be applied to a variety of different forms. In particular, when the pitch of the compound transmission screw is zero, it becomes a fixed transmission.

(2) Based on the screw analysis method of compound transmission, tooth surface and motion process of generator, the classification design method of tooth surface was obtained, including straight tooth, helical tooth, curvilinear tooth and herringbone tooth. By adjusting the tooth profiles, transmission ratio functions, axial displacement functions and structural parameters, different types of SNCG compound transmission can be obtained.

(3) Through five-axis CNC milling, the finished products of SNCG were obtained. Through the tooth surface measurement and transmission experiment, the correctness of the screw analysis method was effectively verified, which laid a theoretical foundation for the further study.

(4) This paper studies the screw analysis method for SNCG compound transmission from the aspect of kinematic geometry. On this basis, the characteristics of bearing and dynamic can be further studied, such as tooth bending stress, contact stress, system vibration and noise, to provide an important reference for the engineering application of SNCG compound motion.

## Supporting information

S1 Data (XLSX)
